# Rhizobial nitrogen fixation efficiency shapes endosphere bacterial communities and *Medicago truncatula* host growth

**DOI:** 10.1186/s40168-023-01592-0

**Published:** 2023-07-03

**Authors:** Beatriz Lagunas, Luke Richards, Chrysi Sergaki, Jamie Burgess, Alonso Javier Pardal, Rana M. F. Hussain, Bethany L. Richmond, Laura Baxter, Proyash Roy, Anastasia Pakidi, Gina Stovold, Saúl Vázquez, Sascha Ott, Patrick Schäfer, Miriam L. Gifford

**Affiliations:** 1 School of Life Sciences, University of Warwick, Coventry, CV4 7AL UK; 2 Warwick Medical School, University of Warwick, Coventry, CV4 7AL UK; 3 Department of Genetic Engineering & Biotechnology, University of Dhaka, Dhaka, Bangladesh; 4 University of Nottingham, Sutton Bonington Campus, Sutton Bonington, Nottingham, LE12 5RD UK; 5 Present Address: Institute of Phytopathology, Research Centre for BioSystems, Land Use and Nutrition, Justus Liebig University, Giessen, 35392 Germany; 6 Warwick Integrative Synthetic Biology Centre, University of Warwick, Coventry, CV47AL UK

**Keywords:** *Medicago truncatula*, Nodulation, Plant–rhizobial interaction, Nitrogen fixation efficiency, Soil, Endosphere, Microbial communities, Nodule sanctioning

## Abstract

**Background:**

Despite the knowledge that the soil–plant–microbiome nexus is shaped by interactions amongst its members, very little is known about how individual symbioses regulate this shaping. Even less is known about how the agriculturally important symbiosis of nitrogen-fixing rhizobia with legumes is impacted according to soil type, yet this knowledge is crucial if we are to harness or improve it. We asked how the plant, soil and microbiome are modulated by symbiosis between the model legume *Medicago truncatula* and different strains of *Sinorhizobium meliloti* or *Sinorhizobium medicae* whose nitrogen-fixing efficiency varies, in three distinct soil types that differ in nutrient fertility, to examine the role of the soil environment upon the plant–microbe interaction during nodulation.

**Results:**

The outcome of symbiosis results in installment of a potentially beneficial microbiome that leads to increased nutrient uptake that is not simply proportional to soil nutrient abundance. A number of soil edaphic factors including Zn and Mo, and not just the classical N/P/K nutrients, group with microbial community changes, and alterations in the microbiome can be seen across different soil fertility types. Root endosphere emerged as the plant microhabitat more affected by this rhizobial efficiency-driven community reshaping, manifested by the accumulation of members of the phylum Actinobacteria. The plant in turn plays an active role in regulating its root community, including sanctioning low nitrogen efficiency rhizobial strains, leading to nodule senescence in particular plant–soil–rhizobia strain combinations.

**Conclusions:**

The microbiome–soil–rhizobial dynamic strongly influences plant nutrient uptake and growth, with the endosphere and rhizosphere shaped differentially according to plant–rhizobial interactions with strains that vary in nitrogen-fixing efficiency levels. These results open up the possibility to select inoculation partners best suited for plant, soil type and microbial community.

Video Abstract

**Supplementary Information:**

The online version contains supplementary material available at 10.1186/s40168-023-01592-0.

## Background

Nitrogen is one of the most essential macronutrients for plant growth. With the challenge of an increasing human population, since the 1950s, food production has benefitted from the development of chemical fertilisers as part of the green revolution to increase crop yields [1], with N being a major component of chemical fertilisers, our agricultural production systems are highly dependent on the synthesis of N fertiliser [2] which has increased 21-fold in the last 80 years, resulting in N fertiliser usage becoming the largest source of agricultural production [3, 4], contributing to increased production of greenhouse gases, and altering ecosystem balance due to nitrogen leaching [5]. Human alteration of the nitrogen cycle is considered to be beyond the acceptable environmental change [6].

Legume plants play a pivotal role in the future of agriculture due to their ability to form a symbiosis with rhizobia, where the bacteria fix nitrogen from the air and make it available for the plant, in exchange for carbon compounds [7]. Previous studies have described that pasture and fodder legumes contribute to the fixation of 12–25 t of nitrogen (N) per year [8]. Legume nitrogen fixation depends strongly on the availability of soil nitrogen and the nitrogen fixation efficiency of the rhizobia they interact with. The more available soil nitrogen there is, the less legumes are likely to invest in rhizobial symbiosis as the latter comes at a higher carbon (C) cost [9]. There are numerous factors that affect the efficiency of nitrogen fixation and other studies have covered those in great detail [10]. Legume symbiosis is an ideal target for use in sustainable agricultural approaches due to the enhancement of soil fertility and the higher independence of these species from nitrogen (N) fertiliser application. Because of this, legumes have largely been used in agriculture, not only for crop production (including soybeans, beans and peas) but also in crop rotation systems and in intercropping, as well as for their contribution to soil health and fertility as winter crops [11]. Recently, it has been proposed to attempt to transfer the ability to fix N to non-legume crops to alleviate the dependence on N fertiliser more widely, either via genetically transforming plants to fix N [12] or via transferring the ability that legumes have to interact with symbiotic rhizobia [3].

Plants recruit microbes as part of their development in the soil environment [13, 14] and this microbial recruitment depends on soil edaphic factors, plant species and genotype [15–17]. Besides rhizobia, legume plants also recruit different subsets of microbial communities depending on their ability to form nodules or not [18, 19]. The design of microbial inoculants that can lead to increased crop production and/or resilience to climate stresses is a promising research direction. However, the design of microbial inoculants, particularly with non-native strains, that work in the field is challenging since the soil microbiome is a complex ecosystem [20]. Work to study inoculation and the persistence of symbionts in complex microbiome ecosystems in a variety of soil types is therefore key.

In nitrogen fixation-based symbiotic systems, efficiency is vital. Different species of rhizobia can interact with the same plant host, leading to very different outcomes across the mutualistic-pathogenic continuum [21, 22]. However, less is known about how different rhizobial inoculants affect not only legume growth and legume molecular responses but also adaptation to different soil environments and microbial recruitment. To understand the influence of the efficiency of legume symbiosis in the host-soil environment, we carried out a large mesocosm experiment to study the symbiosis of the model *Medicago truncatula* and three different N-fixing *Sinorhizobium* strains in representative UK soil types with different fertilisation states. We evaluated the impact of the symbiotic nitrogen fixation efficiency and soil edaphic factors on plant growth, molecular responses, mineral acquisition, and endosphere and rhizosphere microbial recruitment. For this, we used *Medicago truncatula* which is a well-studied model legume at the molecular level, and with which rhizobial strains with different nitrogen fixation efficiency had already been studied [22]. Importantly, the several layers of information that we obtained allowed us to identify specific soil edaphic factors driving the bulk soil microbial community composition that go beyond the classical macronutrients already described in other studies (such as carbon, nitrogen or pH) [23, 24]. As a result, this study advances our understanding of the plant–microbial–soil nexus and is a keystone for future legume microbial inoculant design adapted to specific soil types that will allow host–soil specific fine-tuned sustainable microbiological solutions to be designed for agriculture.

## Results

### *Medicago truncatula* nutrient supply depends on the efficiency of *Sinorhizobium* strains and soil nutrient states

**Fig. 1:**
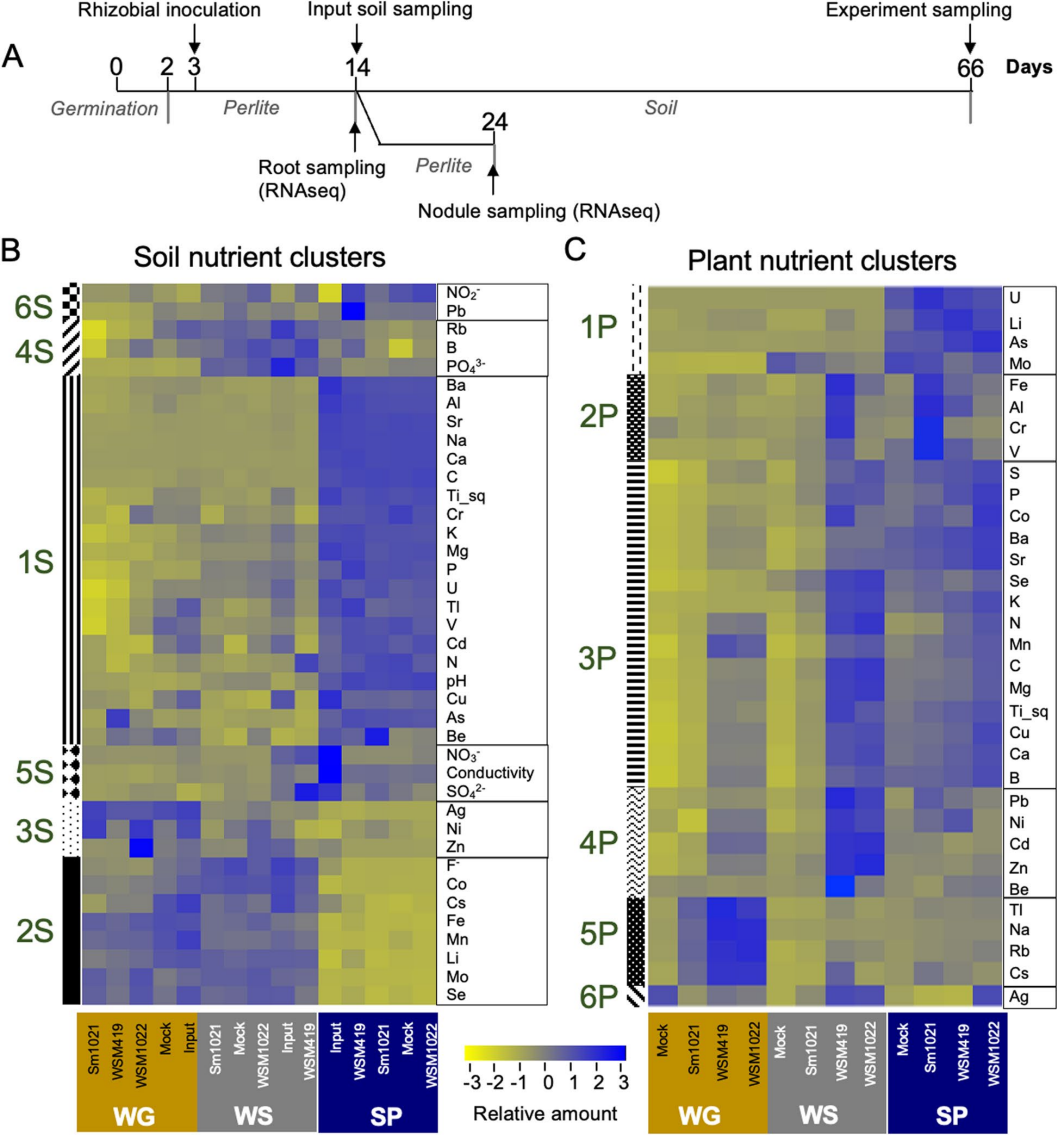
Plant nutrition is affected by a combination of rhizobial inoculant and soil edaphic factors. **A** Experimental design for growth and harvesting of soil, microbe and plant material. Seeds were germinated in plates for 2 days and moved to perlite pots, inoculated 1 day later, then moved to soil 11 days later. Input soil was sampled at the start of the experiment, then aboveground plant, bulk soil and microbiome samples taken at 66 days after germination. RNAseq samples were taken from perlite experiments at 11 and 21 dai. **B** Clustering of bulk soil edaphic factor parameters for the rhizobia-soil type combinations into 6 clusters (1S to 6S); see Supplementary Data S1 for values. **C** Clustering of plant nutrient levels for the rhizobia soil type combinations into 6 clusters (1P to 6P); see Supplementary Data S2 for values; soil types labelled as: WG-ocre, WS-grey and SP-navy (*n* = 4 pooled samples of 8 soil or plant samples for **B** and **C**)

To understand the plant–rhizobia–environment nexus, we asked if the interaction of *Medicago truncatula* with *Sinorhizobium* strains whose nitrogen fixation efficiency varies [22, 25] is also impacted by their soil environment. We set up an experiment to measure plant growth and nutrient content using three rhizobial strains and three soil types with differing levels of nutrients (Fig. 1A). At 3 days after germination, *M. truncatula* roots were mock-inoculated or inoculated with one of three rhizobial strains, low-efficiency *S. meliloti* Sm1021 [22] or the high-efficiency strains *S. meliloti* WSM1022 [25, 26] and *S. medicae* WSM419 [27] in perlite and transferred to soil at 14 days after germination/11 days after inoculation (dai). We included two sandy loams with ~ 2% organic matter (Wharf Ground, WG and Wick Series, WS) and one loam with ~ 2.5% organic matter (Spalding, SP) in our studies as they are representative of ~ 70% of UK arable soil types and because of their different fertilisation statuses (Supplementary Data S1 [Soil_properties]).

The soil was sampled at the beginning of the experiment (day 14) ‘input’ and at the end of the experiment (day 66) ‘bulk’ (Fig. 1A), and we measured pH, conductivity, mineral content by ICP-MS and total C and N levels (Supplementary Data S1). We used hierarchical clustering to group soil edaphic factors according to their profile, finding 6 clusters of nutrients amongst all soil samples (Fig. 1B). The SP soil was enriched for carbon (C), calcium and magnesium and had a higher pH and conductivity (Fig. 1B, cluster 1S, Supplementary Data S1 [C, Ca, Mg and pH]); all rhizobial inoculations resulted in a higher concentration of nitrite (Fig. 1B, cluster 6S, Supplementary Data S1 [Nitrite]). The WS soil was enriched for available phosphate (Fig. 1B, cluster 4S, Supplementary Data S1 [Phosphate]) and both WG and WS soils were enriched compared to SP soil for elements including molybdenum, cobalt, iron and pH (Fig. 1B, cluster 2S, Supplementary Data S1 [Mo, Co, Fe]).

Soil edaphic factors for input (pre-experiment) and bulk (post-experiment) soil clustered together per soil type (all samples from one soil type clustered together on the vertical axis), showing that neither mock inoculation, rhizobial inoculation or the presence of plants led to a global change them over the course of the experiment (Fig. 1B). There were only a few elements whose abundance varied over the experiments; nitrate concentration and conductivity (a reflection of soil solution ion concentration) which had lower values at 66 vs. 14 days, particularly in SP and WS soils (Fig. 1B, cluster 5S, Supplementary Data S1 [Conductivity and Nitrate]). This is likely a result of nitrate being used up by plants in these soils. The fact that this does not seem to occur in WG soil is likely due to the comparative low input soil nitrate level. Plants were watered with reverse osmosis water (ions were not added), thus over time ionic uptake from soil by the plant is consistent with the reduced conductivity observed.

When clustering the nutrient profiles of aboveground plant material ‘shoots’, we found 6 clusters that linked soil nutrition and rhizobial efficiency to plant nutrient content (Fig. 1C). These show that different strains vary in their ability to increase plant nitrogen supply and affecting other nutrient content (e.g. carbon) and that this is affected by and limited by abundances in the soil environment. For example, plants in symbiosis with high-efficiency rhizobial strains on WG and WS soils were able to increase macro- and micronutrient uptake, when compared to mock or plants in symbiosis with low-efficiency Sm1021, independently of the profile of those elements in the input soil (Fig. 1C, Supplementary Data S2). In general, differences in growth across soil types including in high-efficiency symbiosis are most likely due to limitation of one (Liebig’s law of the minimum) or several [28] nutrients in the input soil. For example, plants grown in WS soil have access to more phosphate than in SP, but total P *in planta* is not different between SP and WS plants. This points to the uptake of P being limited by a different element (or condition) in WS soil. High-efficiency rhizobial symbiosis led to proportionally greater accumulation of sodium in shoots of plants on WG soil than the higher-sodium SP soil, despite soil Na being much higher in SP (Fig. 1C, cluster 5P, Supplementary Data S2 [Na]), and there was a greater accumulation of Zn in WS-grown plants compared SP-grown plants (Fig. 1B, Supplementary Data S2 [Zn]), despite the lack of difference in soil Zn (Fig. 1C, cluster 4P, Supplementary Data S1 [Zn]).

**Fig. 2:**
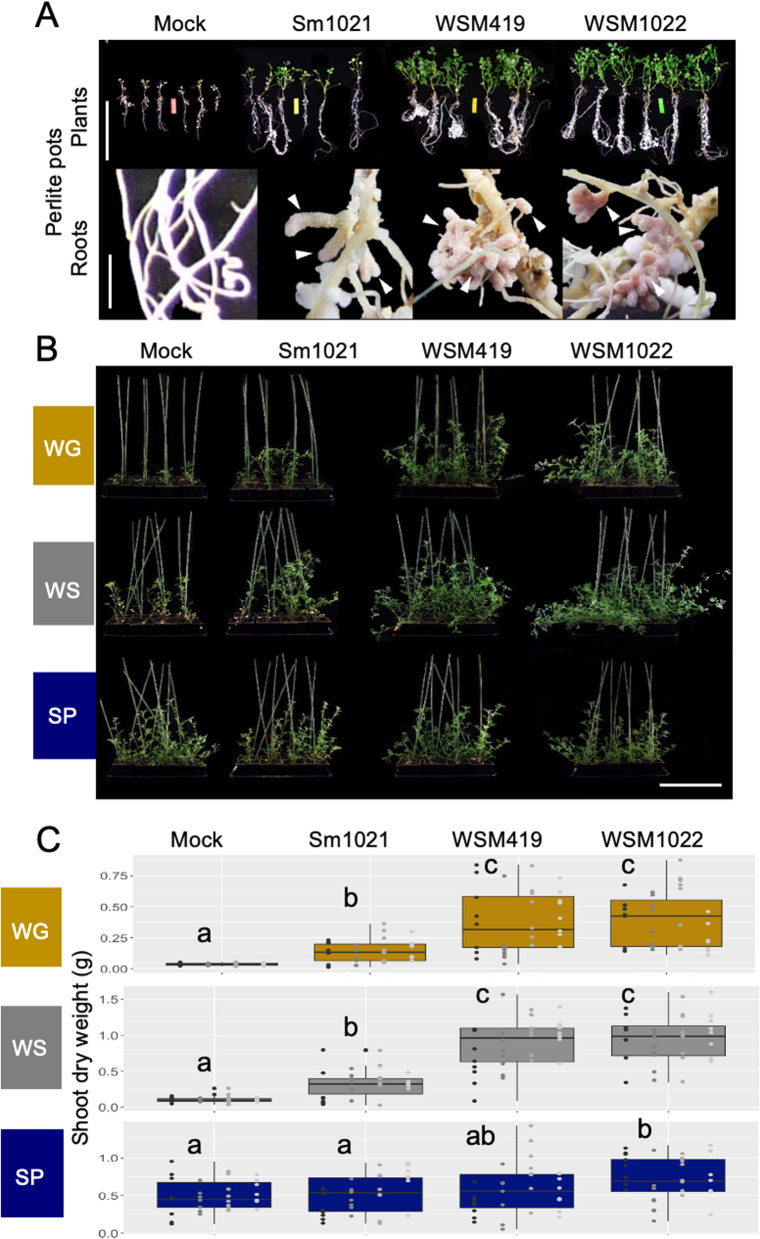
Plant biomass and nutrition are affected by a combination of rhizobial inoculant and soil edaphic factors. **A** Inoculation with high-efficiency rhizobial strains WSM419 and WSM1022 lead to plants with a larger biomass and more-developed nodules. White arrowheads point to nodules. Plants were inoculated with rhizobia and grown in perlite pots for 5 weeks; whole plants and roots are shown; scale bar = 10 cm for plants and 2 cm for roots. **B** After 66 days, the soil, rhizosphere and endosphere of plants inoculated with rhizobial strains (or mock) grown in WG/WS/SP soil were harvested. Representative plant growth is shown; scale bar = 10 cm. **C** Boxplots of dry weight values for plants in **B**; letters denote significantly different values according to ANOVA and TukeyHSD, *P* < 0.05; see Supplementary Data S3 for plant dry weight values and statistical analysis (*n* = 32, 8 biological replicates, indicated with individual points, from 4 experimental replicates in different shades of grey)

Inoculation with high-efficiency rhizobial strains resulted in a significant increase in dry weight and shoot size (*P* < 0.05) compared to mock-inoculation (Fig. 2, Table S1), and this could also be seen in increased C content (Fig. 1C, Supplementary Data S2 [Carbon]). In the SP soil, only WSM1022 inoculation increases dry weight compared to the mock and Sm1021 inoculations (Fig. 2C). This is, most likely, driven by generally higher macronutrient levels (including N) in this soil compared to the other two soils (Fig. 1B, cluster 1S, Supplementary Data S1 [N]). The higher soil N levels might lead to plants being less symbiotically active, even with high-efficiency strains in the SP soil, and therefore even the significant differences are less pronounced in this soil (Fig. 2C). Comparing only the mock inoculations, plants in the WG soil are the smallest, most likely due to this soil significantly lower levels of key nutrients N and P compared to the other soil types (Supplementary Data S1 [N and P]).

The increase in dry weight was seen irrespective of soil nutrition in the WG and WS soils (Fig. 2C, Table S1) which correlated with increased shoot content of many macronutrients including C, N, P, K and Ca (Fig. 1C, cluster 3P, Supplementary Data S2 [C, N, P, K and Ca]) (Fig. 2C). Interestingly, even in SP soil, the high-efficiency strain WSM1022 led to increased dry weight (Fig. 2C, Table S1) when compared to mock or Sm1021-inoculation; however, this cannot be linked to a significant increase of any specific nutrient (e.g. carbon content in WSM1022-inoculated plants in the SP soil is not significantly different from mock-inoculated plants in this soil (Supplementary Data S2 [Carbon]).

The key nutrient molybdenum (Mo), which is required for the correct functioning of the nitrogenase complex in nodule symbionts [29], was highly abundant in shoots of plants grown on SP soil (Fig. 1C, cluster 1P, Supplementary Data S2 [Mo]), despite SP soil having less Mo than the other two soils (Fig. 1B, Supplementary Data S1 [Mo]). However, Mo concentration in the shoot was lower in WS plants nodulated by the high-efficiency WSM1022 compared to mock-inoculation (Fig. 1C, Supplementary Data S2 [Mo]), suggesting that in higher-efficiency nodules, Mo might be mobilised from the soil, but not moved to shoots. The greater Mo content in shoots of plants grown in the richer SP soil could be due to reduced requirement of nodulation.

Overall, we found that rhizobial inoculation improved mobilisation of nutrients to the shoot which correlated with increased plant growth and that this was dependent on the N fixation efficiency of the rhizobial strain and the soil nutrient content. Moreover, high-efficiency strains improve nutrient uptake even in high nutrient soils such as SP, and the WSM1022 inoculum can also improve plant dry weight.

### Specific soil edaphic factors determine soil microbial community structure

We next asked to what extent the communities of microbes were correlated with different soil types with differing nutrient contents and the effect of the WSM1022 inoculation. After profiling the bacterial and fungal communities in input and bulk soil via sequencing, we integrated this data with our quantitative data on soil nutrient elements using a canonical correspondence analysis (CCA). CCA allows a simultaneous visualisation of the explanatory variables determined from response data (the microbial communities) as a linear combination of measured predictors (soil parameters) [30]. We focussed on evaluating the impact of the high-efficiency strain WSM1022 vs. mock since WSM1022 had the greatest impact on plant yield independently of soil type. Soil edaphic factors, rather than the experiment or the rhizobial strain used for inoculation, defined both bacterial and fungal soil communities based on comparing beta diversity (Fig. S1B and Fig. S2B) and there were no significant differences (DESeq2 enrichment *P* < 0.05, Supplementary Data S3 [Exp1BactsoilDiff & Exp1FungiSoilDiff]) in amplicon sequence variants (ASVs) in bulk soil between mock and WSM1022 in any soil type. This lack of global effect in bulk soil according to rhizobial inoculation was also seen when examining alpha diversity for bacterial (Fig. S1A) and fungal (Fig. S2A) communities. Therefore, we then analysed the correlation of the microbial community structure to the soil edaphic factor clusters.

**Fig. 3:**
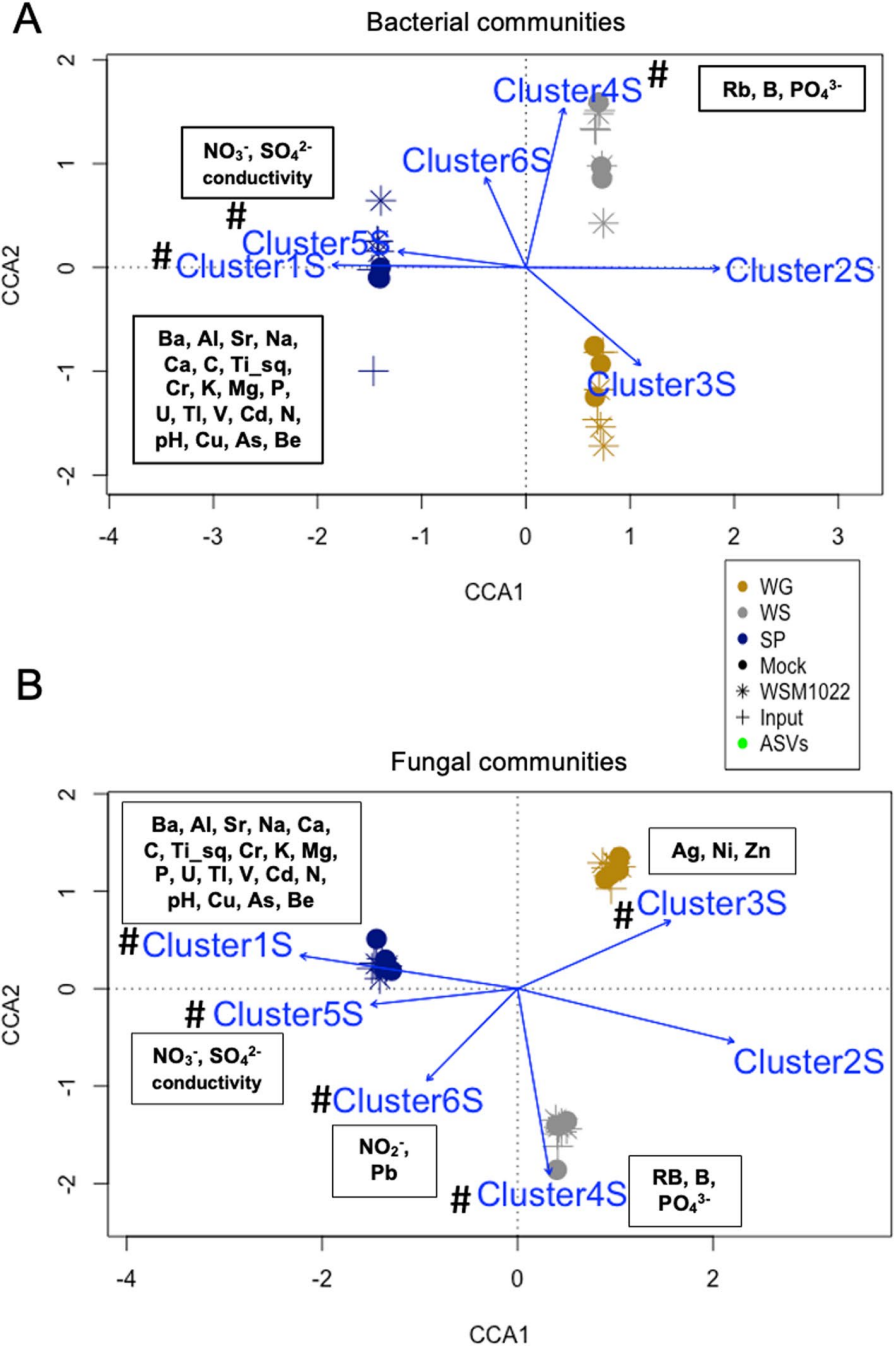
Soil microbial communities are shaped by specific soil edaphic factors.** A** CCA for input soil bacterial communities with the clusters of soil edaphic factors from Fig. 1B (blue arrows indicate the direction and magnitude (length) of the correlation), boxes with soil properties/elements are included for those clusters that have a significant correlation; hash symbols denote significant correlation. **B** CCA for input soil fungal communities with the clusters of soil edaphic factors from Fig. 1B (blue arrows indicate the direction and magnitude (length) of the correlation), boxes with soil properties/elements are included for those clusters that have a significant correlation; hash symbols denote significant correlation (*P* < 0.05) permutation test (1000 permutations) of CCA fit by vegan R package. Individual plot points indicate pooled samples of 8 biological replicates

For bacterial communities, soil type explained 60.4% of the variation in community structure (*P* < 0.01, Supplementary Data S3 [Exp1BactBetaStats]and Table S2), and the SP soil community clustered distinctly from the WG and WS soils in principal component 1 (PCo1) (Fig. 3A, Fig. S1B). Based on the CCA, it was found that bacterial communities could be distinguished per soil type. This was principally by clusters 1S and 5S that had significantly higher values of C, Ca, Mg as well as higher pH in SP soil than WG and WS, as well as cluster 4S that had higher values of phosphate in the WS soil than WG and SP (Fig. 3A, Supplementary Data S1 [Phosphate]). Based on ASV analysis, bacterial communities were dominated by the phyla Actinobacteria and Proteobacteria across all soil types, independently of the rhizobial inoculation (Fig. S1C).

For fungal communities, which were dominated by the Sordariomycetes class, soil type was again the key shaping factor, explaining 78.7% of the variation in community diversity changes (*P* < 0.01, Fig. S2B, Supplementary Data S3 [Exp1FungiBetaStats] and Table S2). Fungal communities were distinguished by higher values of macronutrients and pH in SP soil (clusters 1S and 5S, Fig. 3B, Supplementary Data S1). In this case, the WS and WG soils could also be distinguished (Fig. 3B in CCA2 and Fig. S2B in PCo2), potentially related to the higher abundance of phosphate in WS soil compared to WG (Fig. 1B, Cluster 4S, Supplementary Data S1). Thirty fungal ASVs were more abundant in WS soil, and four of them followed the same accumulation pattern as phosphate (WS > SP > WG, Supplementary Data S3 [Exp1FungiSoilDiff]); amongst these were two *Fusarium* ASVs. [31] In addition, cluster 3S which had higher values of zinc in WG soil was a key differentiator for WG soil fungal communities (Fig. 3B, Supplementary Data S3 [Exp1FungiSoilCCA]). Interestingly, significant differences in nitrite levels in cluster 6S correlate with fungal community structure differences in the SP soil (Fig. 3B, Fig. S2D, Supplementary Data S3 [Exp1FungiSoilCCA]).

In summary, when comparing edaphic factors with the overall microbiome communities, macronutrients and pH were found to be key potential shapers, but also, less well understood soil parameters such as zinc levels played a major role.

### Rhizosphere bacterial communities are defined by both soil type and rhizobial inoculation

**Fig. 4:**
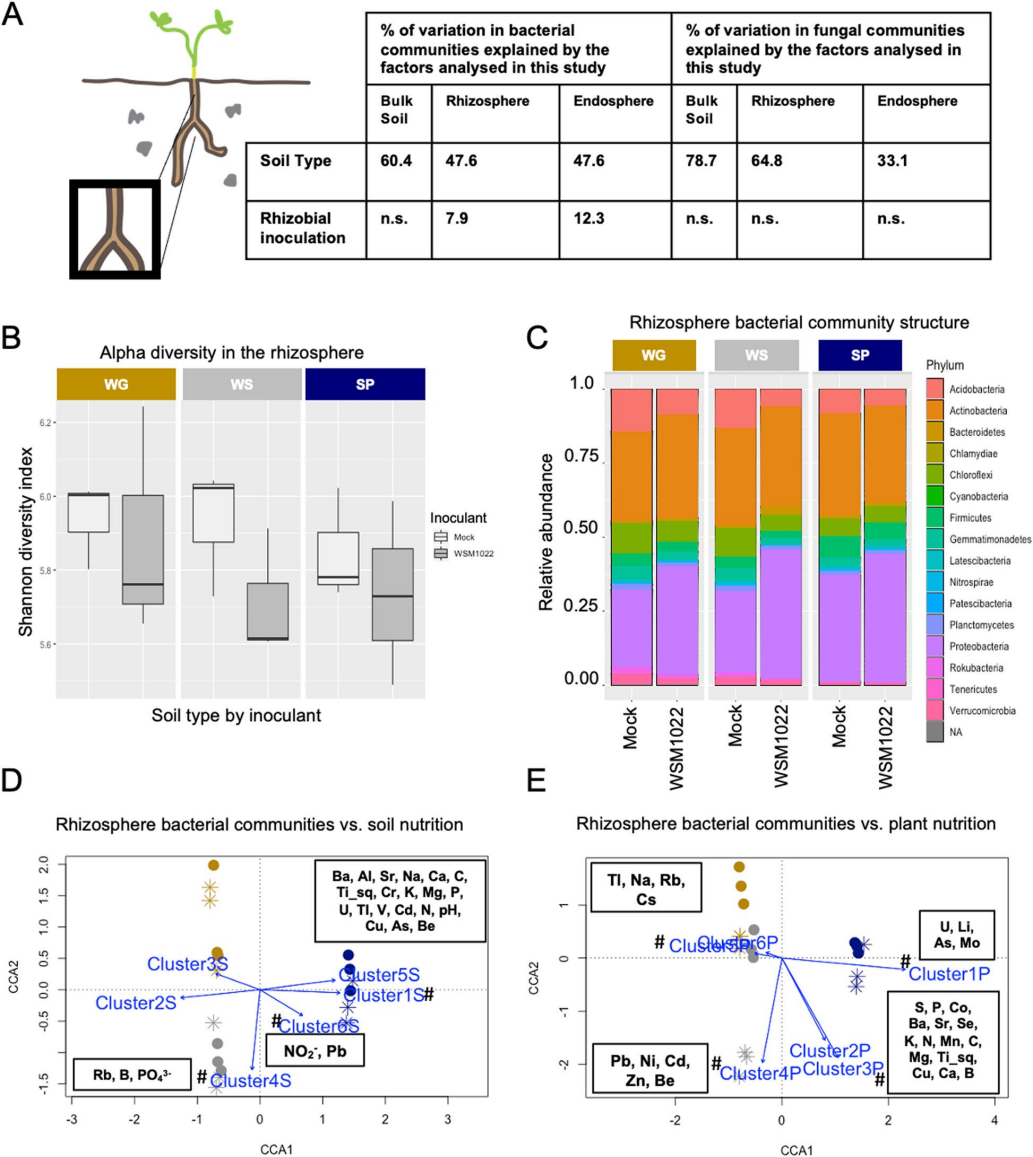
Rhizosphere bacterial communities are shaped by rhizobial inoculant, impacting plant nutrition. **A** Global percentage of variation in microbial communities explained by the factors studied in this study. **B** Alpha diversity in rhizosphere bacterial communities. **C** Dominant rhizosphere bacterial taxa across rhizosphere samples, average relative abundance of taxonomic groups making up > 0.1% of total abundance; each bar is the average of 3 pooled samples of 8 biological replicates. **D** CCA of beta diversity of rhizosphere bacterial communities in rhizosphere soils from mock and WSM1022 inoculations, with the clusters of soil edaphic factors from Fig. 1B (blue arrows indicate the direction and magnitude (length) of the correlation). **E** CCA of beta diversity of rhizosphere bacterial communities in rhizosphere soils from mock and WSM1022 inoculation, with the clusters of plant shoot nutrients from Fig. 1C (blue arrows indicate the direction and magnitude (length) of the correlation); for **D**, **E** hash symbols denote significant correlation (*P* < 0.05) permutation test (1000 permutations) of CCA fit by vegan R package. Individual plot points indicate pooled samples of 8 biological replicates

We next asked how nodulation might shape microbial communities directly around the root (rhizosphere) by profiling these samples for the high-efficiency interaction with WSM1022 vs. mock-inoculated plants. Whilst no variation was significantly explained in the beta diversity analysis for fungal communities (Fig. 4A), the bacterial community was significantly reshaped within this compartment. For both bacterial and fungal communities, soil type explains a much higher percentage of microbial community composition than inoculation strain (Fig. 4A). In the rhizosphere, we found that both the soil type and the rhizobial inoculant defined the variation in bacterial community structures, based on alpha diversity (Fig. 4B) and CCA (Fig. 4D–E). Soil type explained 47.6% of the variation (*P* < 0.01) whilst rhizobial inoculant explained 7.9% (*P* < 0.05, Fig. 4A, Supplementary Data S3 [Exp1BactBetaStats]).

The major axis of variation (CCA1) separated the SP soil from the WG and WS soils, explaining 14.4% of the variance whereas the second axis (CCA2) explained 7.9% of the variance (Fig. 4D). Interestingly, rhizobial inoculation shifted the bacterial community structure in the same direction for all soil types, visible on the CCA2 axis, suggesting a common impact of rhizobial inoculation (Fig. 4D).

To ask whether soil edaphic factors or aboveground mineral composition correlated with rhizosphere bacterial communities, we used CCA, finding that soil clusters associated with the SP soil still explained the largest amount of variation in bacterial communities (Fig. 4D: clusters 1S and 6S explain 11.4% (*P* < 0.001) and 7.8% (*P* < 0.01) of variance, respectively). The phosphate cluster 4S still characterised WS soil bacterial communities in the rhizosphere (Fig. 4D) but when comparing rhizosphere profiles to plant mineral content, we found other connections, such as the high plant shoot Mo cluster in SP plants aligning with the SP rhizosphere samples (Fig. 4E, cluster 1P). Interestingly, the CCA also revealed plant nutrient clusters that correlate with the rhizosphere bacterial communities, only after inoculation with rhizobia. For example, whilst the large macronutrient cluster (3P) was found to align with all SP rhizosphere samples, it also aligned with the rhizobial-inoculated WS rhizosphere samples (Fig. 4E, cluster 3P). The high Zn cluster (cluster 4P) aligned with rhizobia-inoculated WS and rhizobia-inoculated WG rhizosphere samples (Fig. 4E, cluster 4P) and the high Na cluster aligned with rhizobia-inoculated WG rhizosphere samples (Fig. 4E, cluster 5P, Fig. S3). These correlations indicated that changes in the rhizosphere as a combinatorial result of soil edaphic factors and rhizobial inoculation impact plant nutrition outcome.

### Endosphere bacterial community is shaped by nitrogen fixation efficiency during rhizobial symbiosis

**Fig. 5:**
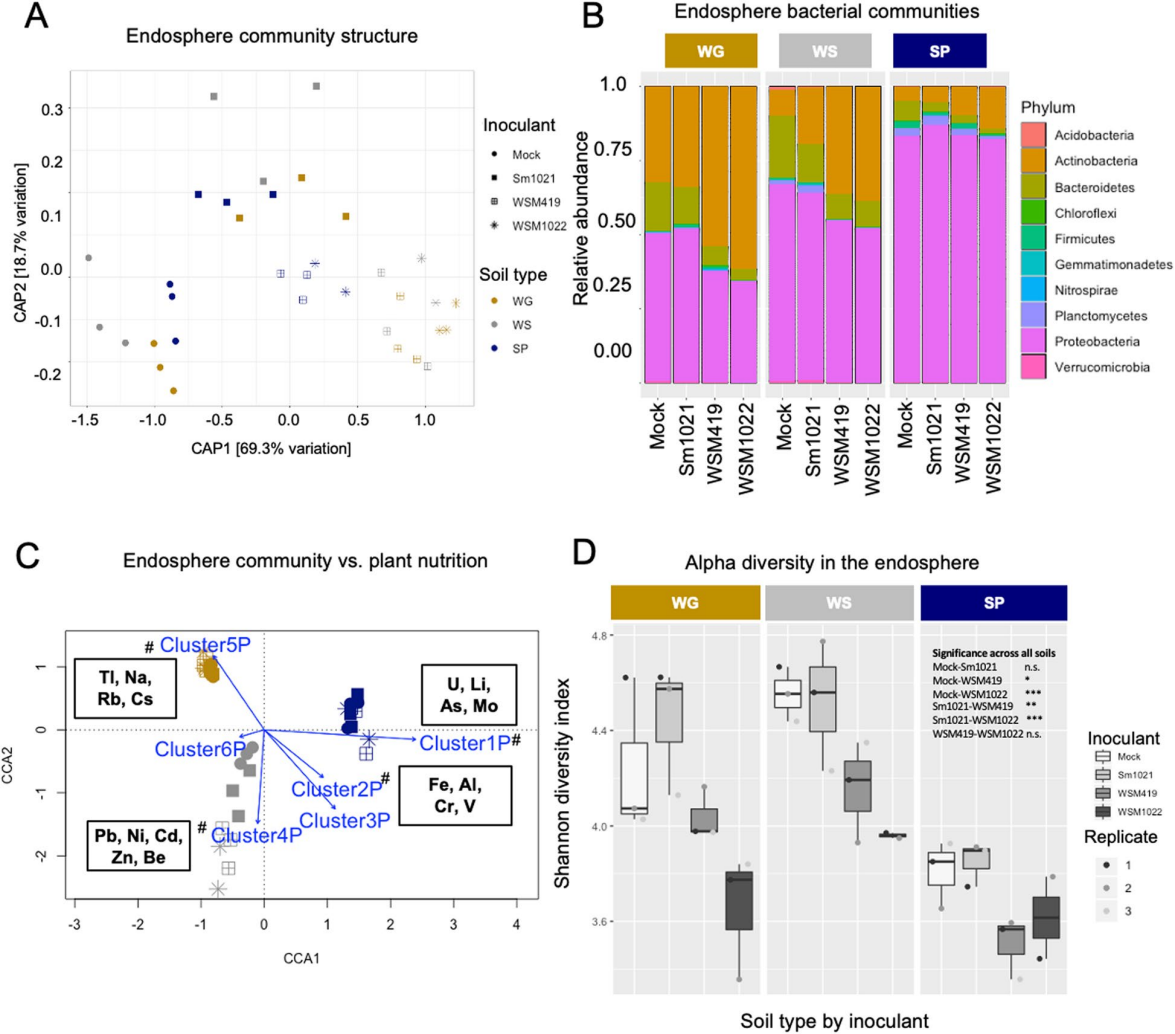
Endosphere bacterial community analysis and correlation with specific aboveground minerals. **A** Inoculation-constrained canonical analysis of principal coordinates of the bacterial community structure in the different inoculations and soil types. **B** Dominant endosphere bacterial taxa. **C** CCA analysis for endosphere bacterial communities with the clusters of plant shoot nutrients from Fig. 1C (blue arrows indicate the direction and magnitude (length) of the correlation); hash symbols denote significant correlation (*P* < 0.05) permutation test (1000 permutations) of CCA fit by vegan R package. **D** Alpha diversity in endosphere bacterial communities. Individual plot points indicate pooled samples of 8 biological replicates. Significant differences are indicated for inoculation/treatment comparisons across all soil types ANOVA TukeyHSD (*P < 0.05, **P < 0.01, ***P < 0.001)

Since the largest inoculation effect was on the endosphere bacterial community (Fig. 4A), to distinguish the impact of rhizobial inoculation on the plant endosphere communities, we performed a rhizobial strain-constrained analysis of principal coordinates (CAP) of the microbial communities from plants that were Sm1021, WSM419, WSM1022, or mock-inoculated (Fig. 5A). This is a PCoA analysis that represents only the variation associated with rhizobial strains. We were able to distinguish changes to the bacterial community that are distinct between high-efficiency and low-efficiency rhizobial inoculation (Fig. 5A) and found that inoculation with higher efficiency rhizobial strains led to greater differences along axis CAP1 (which represents 69.3% of constrained variation, Fig. 5A).

**Fig. 6:**
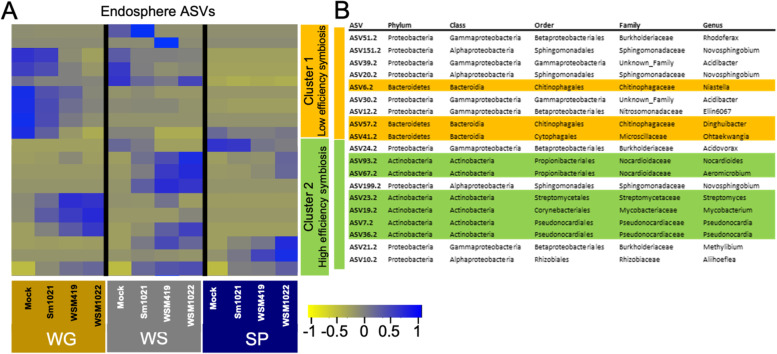
Endosphere bacterial community analysis and correlation with specific aboveground minerals. Heatmap (**A**) and table (**B**) showing normalised abundance of significantly enriched ASVs according to DEseq2 analysis, with Bacteroidetes present only in cluster 1, which tends to have reduced abundance during high-efficiency symbiosis, and Actinobacteria present only and enriched in cluster 2 which tends to have increased abundance during high-efficiency symbiosis. Scale represents a z-score calculated per ASV

Endosphere bacterial communities were dominated by the phyla Actinobacteria and Proteobacteria across all soil types independently of the rhizobial strain (Fig. 5B), yet an alteration in community composition and a shift in plant nutrition upon rhizobial inoculation and their correlation could be observed (Figs. 1C and 5C). Remarkably, species diversity in the endosphere in high-efficiency rhizobia-inoculated plants was lower than that of low-efficiency rhizobia-inoculated plants for all soil types (Fig. 5D, Supplementary Data S3 [Exp2BactAlphaStats]). This suggested that inoculation of plants with high-efficiency rhizobial symbionts might lead to more selective recruitment endosphere microbiome assemblies. We used DESeq to test for differential abundance of ASVs between rhizobia-inoculated samples and mock inoculation. These were grouped into two clusters showing, generally, either increased or decreased abundance relative to symbiosis efficiency (Fig. 6). Increased abundance and diversity of Actinobacteria phyla were present and also enriched (× 1.9) compared to other ASVs exclusively during high-efficiency symbiosis (hypergeometric *P*-value = 0.017; Fig. 6, cluster 2). Similar significant differences were not found for fungal communities (Fig. S4).

### Nitrogen fixation efficiency shapes the nodule transcriptome

**Fig. 7:**
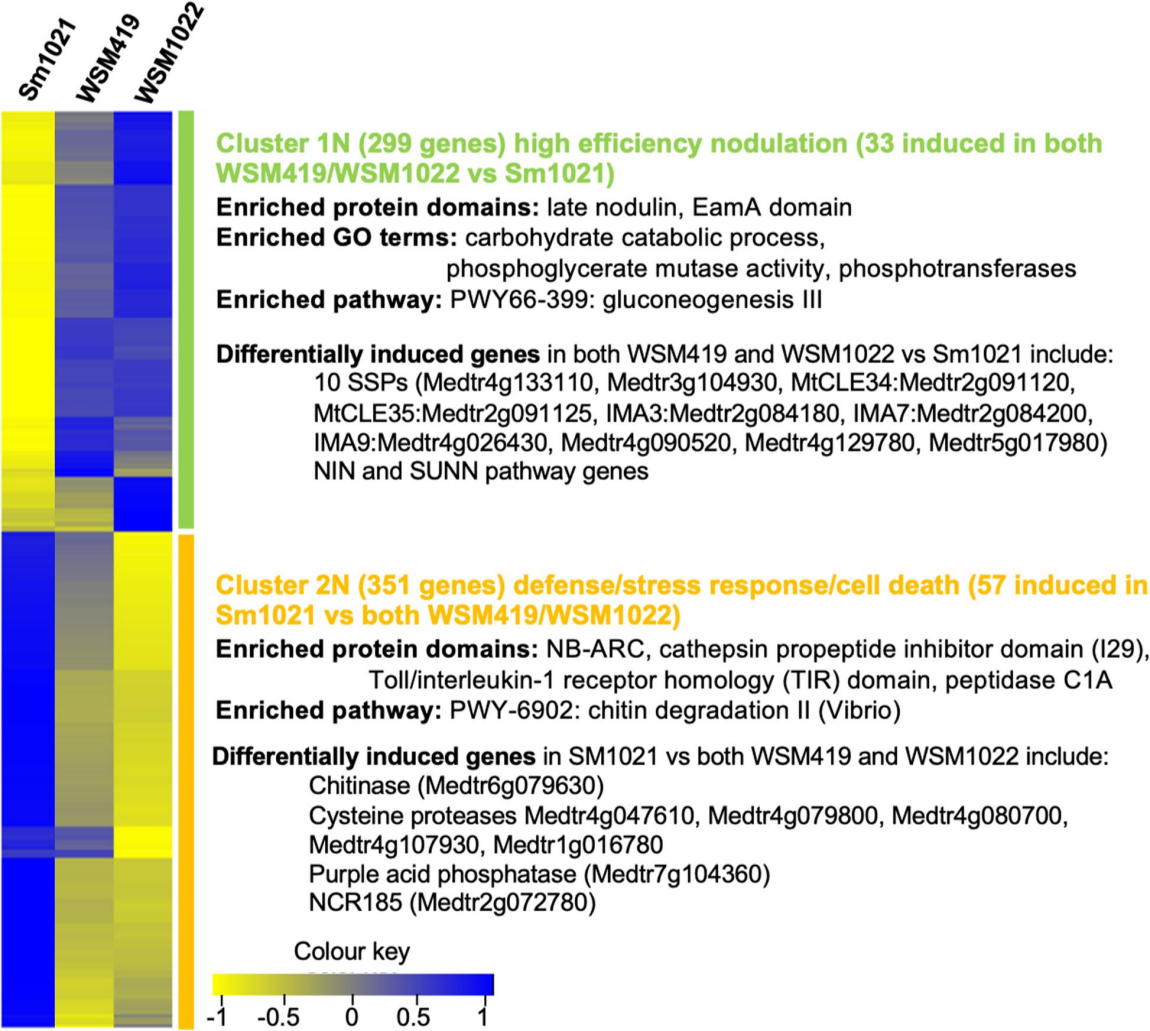
Genes that are differentially expressed in nodules vary depending on rhizobial symbiont. Heatmap of differentially expressed (*P* < 0.05 DEseq2 R) transcripts between nodule samples with analysis of GO term, protein domain and pathway enrichment (*n* = 2)

Since the endosphere bacterial community structure and plant nutrition were dependent on the rhizobial inoculant, we measured plant root and nodule transcriptomes to pinpoint how strain efficiencies translate into changes in plant gene expression that underpin the dramatic phenotypes that we confirmed both in perlite and soil (Fig. 2). We sampled nodules from plants grown in perlite at 21 days after inoculation (dai) as well as roots 11 dai for RNAseq analyses (Table S3). In nodules, we detected 650 differentially expressed genes (DEGs) between either of the high-efficiency strains (WSM1022, WSM419) and the low-efficiency strain (Sm1021), which could be separated into two clusters (Fig. 7). Subcluster 1N, with 299 genes, was expressed more highly in highly efficient nodules. Of those 299 genes, 33 were differentially expressed in both WSM1022 and WSM419 (sub-cluster 1N, Fig. 7, Table S3). With higher expression in low-efficiency nodules, subcluster 2N contains 351 genes, of which 57 were differentially expressed in in both WSM1022 and WSM419 (sub-cluster 2N, Fig. 7, Table S3). To identify differences based on efficiency of nitrogen fixation, we focused our analysis in these sub-clusters. Notably, both sub-clusters contain several genes known to be involved in the regulation of nodulation. Subcluster 1N is populated with genes related to nodule amino acid export, iron and molybdenum uptake, cytokinin biosynthesis and small regulatory peptides indicating successful nodulation, with sub-cluster 2N-containing genes are linked to defence, senescence and termination of symbiotic partnership.

Sub-cluster 1N includes Medtr0542s0020, an ortholog of the Arabidopsis glutamine dumper protein that has been shown to export amino acids from cells [32]. This activity is most likely required after rhizobia-derived ammonium is assimilated into glutamine and asparagine to distribute N around the plant [33] and higher expression of this gene is likely a reflection of higher N fixation. MtIPT3 (Medtr1g072540), which is involved in the synthesis of cytokinins, that are well known nodule development regulators [34], *MtMOT1.3* (Medtr3g464210), the only Medicago nodule-specific molybdate transporter [35], and the nicotianamine synthase (Medtr1g084050) which is key to N fixation efficiency [36] are all found in this sub-cluster.

Interestingly, SSPs were over-enriched 3.47 times (*P* < 0.001) in sub-cluster 1N (Fig. 7). These genes are involved in many different plant growth and development processes and in *Medicago truncatula*, they have specifically been linked to the nodulation process [37, 38]. In contrast, we only found three SSPs (5.3% of 57 genes) in sub-cluster 2N (Medtr8g030733, Medtr7g012350, Medtr8g031150), suggesting that SSPs could be largely linked to more efficient nodulation partnerships. Nodule-specific cysteine-rich peptide NCR185 (Medtr2g072780) was upregulated in subcluster 2N. *Nodulation inception* (*MtNIN* (Medtr5g099060) plays a key role during nodulation and *supernumerary nodules* (*SUNN*) regulates the autoregulation of nodulation (AON) pathway. We asked to what extent higher or lower efficiency nodulation utilises defined NIN-regulated genes [39] or defined SUNN*-*regulated genes [40]. We found that sub-cluster 1N contains 17 NIN-induced genes and 1 NIN-repressed gene whereas sub-cluster 2N has 10 NIN-induced genes and 25 NIN-repressed genes. In sub-cluster 1N, there is one SUNN-repressed gene and 7 SUNN-induced genes, and in sub-cluster 2N, there are 10 SUNN-repressed genes and 5 SUNN-induced genes. NIN can act as a positive or negative regulator of nodulation [41] since *nin* mutants cannot form successful symbiosis but NIN has also been implicated in the CLE35/34, SUNN-mediated onset of AON. These results suggest that both NIN- and SUNN-induced pathways are more active in sub-cluster 1N than in sub-cluster 2N. Since our experiments were carried out without any external nitrogen input, the expression of these genes in highly efficient nodules can only be linked to internal nitrogen levels (higher nitrogen fixation efficiency) and suggests that AON has been initiated in highly efficient nodules. In contrast, in sub-cluster 2N, pathways relating to NIN and SUNN seem to be suppressed and plants could still be able to initiate new nodules. Within the low-efficient symbiosis sub-cluster 2N is the NIN-induced nitrate transporter Medtr8g069775, suggesting an increased demand for nitrate due to reduced amounts of rhizobia derived-N in these nodules.

In both subclusters, there are genes related to defence responses (Fig. 7). In sub-cluster 1N, there are five homologs of genes found to be negatively regulated by successful fungal pathogen infection in alfalfa (Medtr1g084050, Medtr4g133110, Medtr1g072540, Medtr4g081130, Medtr3g104930 [42]); all except Medtr4g081130 (which was down regulated in resistant plants) were similarly downregulated in susceptible alfalfa [42]. In sub-cluster 2N, there is a much higher representation of genes involved in stress and degradation compared to sub-cluster 1N. There are five cysteine protease-associated genes (Medtr4g047610, Medtr4g079800, Medtr4g080700, Medtr4g107930, Medtr1g016780) and a further peptidase (Medtr4g093820). Some of these proteins are known to be associated with nodule senescence [43–45]. There is also a chitinase (Medtr6g079630) and a purple acid phosphatase (Medtr7g104360); genes which are specifically known to be early markers of nodule senescence [46]. In sub-cluster 2N, we find one gene downregulated in resistant plants (Medtr1g073990) and nine upregulated in susceptible plants (Medtr7g020980, Medtr0428s0030, Medtr8g030733, Medtr1g062590, Medtr1g080800, Medtr3g104750, Medtr6g007770, Medtr2g009270, Medtr2g040530). Two of these (Medtr1g062590, Medtr1g080800) are known Medicago pathogenesis-related (PR) proteins [47, 48]. We also find Medtr6g088805 and Medtr8g099030 upregulated in sub-cluster 2N, whose Arabidopsis homologs, ADS1 and RBOHF, respectively, are known to be involved in early immune responses during pathogen infection [49, 50]. Overall then, there seem to be a number of differentially expressed genes related to pathogen-like response processes associated with low-efficiency symbiosis.

In contrast to the significant re-programming in inoculated nodules that varies by rhizobial strain, in 11 dai roots, despite there being 2545 DEGs when comparing rhizobial-inoculated and mock-inoculated plants, there were no significant differences between Sm1021 and both WSM419- and WSM1022-inoculated roots. The rhizobial vs. mock DEGs can be separated into two clusters. Cluster 1 with 2050 DEGs includes genes related to early nodulation processes such as nodulins, leghaemoglobins, defence response genes and genes related to CLE peptide-arabinosilation modification (Fig. S5 cluster 1R, Table S3). Amongst these, there are key early regulators of nodulation including *MtNIN* and *MtRPG* (Medtr1g090807), indicative of the activation of nodulation. The induction of *NIN* in roots inoculated with all rhizobial strains has a different effect in the nodule pathways activated or inhibited by it depending on the rhizobial strain as seen in the nodule gene analysis. Cluster 2, with higher expression in mock-inoculated plants which had been grown under nitrogen-limitation contained genes related to stress responses and flavonoid biosynthesis (Fig. S5).

In summary, core nodulation pathways are commonly regulated in roots, despite variations in rhizobial efficiency, but nodules involved in different nitrogen fixation efficiency symbiosis show distinct transcriptional responses.

## Discussion

In this work, we characterised the impact of rhizobial strains with different nodulating and N fixation efficiencies on plant growth, nutrition, host transcriptional changes and microbiome recruitment in distinct and agriculturally relevant soil types. We found that clusters of edaphic factors correlate with soil microbial community structure, with P particularly associated with shaping fungal communities (cluster 4S in Figs. 1B and 3B). For bacterial communities, the SP soil community clustered distinctly from the WG and WS soils which seems likely to be a consequence of the distinct macronutrient and pH levels in SP soil that are known to play major roles in ecosystem shaping; some specific soil edaphic factors, such as pH, C, N or extractable P have been correlated with bacterial or fungal community structure in the past [23, 51, 52]. It has been found that phosphate environment adaptability is a key factor for *Fusarium* fungi to colonise plant tissues [31], suggesting the proliferation of fungal species was highly dependent on P in our experiment. Beyond these well-described factors, we also identified specific and, so far, less well-characterised edaphic factors, which correlated with fungal community structure. Firstly, zinc, whose toxicity has been related to a decrease in bacterial diversity in agricultural soils [53]. In this study, we used soils with non-toxic (average of 62 mg/kg) Zn levels and we found that Zn may play a role in shaping fungal structure (Fig. 3B). Secondly, the finding that that nitrite levels correlate with soil fungal communities (Fig. 3B) is of high significance due to the human influence on global N cycles and the relevance of microbes in shaping these global cycles [54]. The finding that Zn and nitrite levels also correlate with, and help explain variability in, fungal community structure points to a potential role for them in shaping microbial communities.

**Fig. 8:**
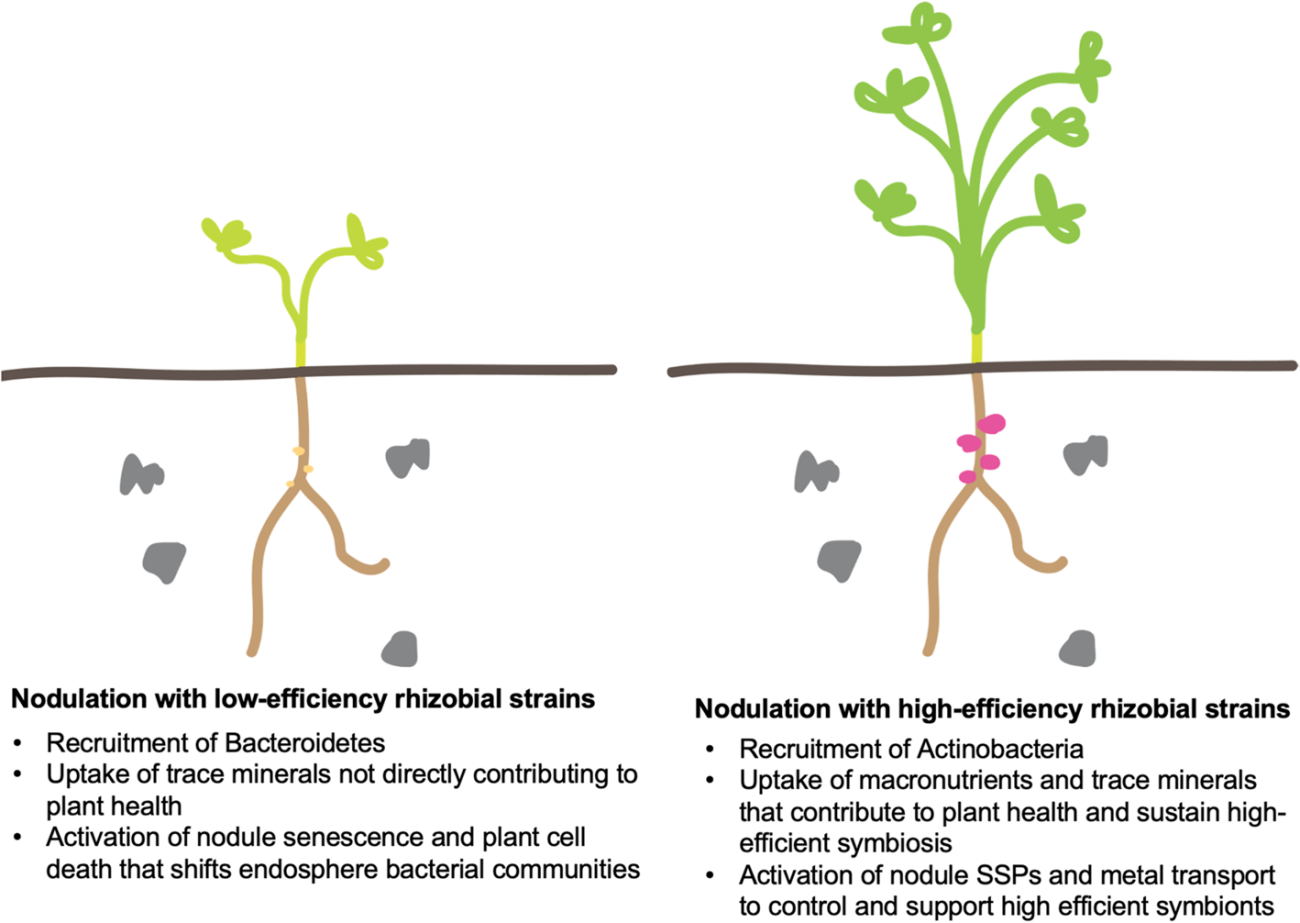
Graphical summary of the rhizobial–plant–soil mechanisms uncovered by this work. Rhizobial nitrogen fixation efficiency impacts endosphere bacterial community structure, nutrient uptake and delivery of carbohydrates and metals to symbionts in *Medicago truncatula*

For both bacterial and fungal communities, soil type explains a much higher percentage of microbial community composition than inoculation strain (Fig. 4A). Although this percentage is highest in bulk soil fungal community composition (78.7%), this substantially decreases towards the endosphere to 33.1%. This reduction is far less drastic in bacterial communities, from 60.4% in bulk soil to 47.6% in the endosphere. This suggests that the fungal community structure is less dependent on soil type than the bacterial community. Endosphere fungal community structure might be influenced by currently unknown factors in the soil, such as microbe–microbe interactions [55] or metabolites not measured in this study. Contrastingly, rhizobial inoculation has a higher impact in bacterial community structure in the endosphere, explaining 12.3% in this compartment and a reduced 7.9% in the rhizosphere (Fig. 8). In contrast with previous findings in the widely studied *Arabidopsis thaliana* [56], we did not observe this effect in fungal communities, suggesting that bacterial endophyte inoculation might have a more specific effect in legume plants.

Endosphere fungal community structure was not significantly affected by rhizobial N fixation efficiency (Fig. S4). However, only 33% of the variation was explained by soil type (Fig. 8, Supplementary Data S3 [Exp2FungiAlphaStats] and Table S3). Bulk soil fungal alpha diversity decreased with soil fertility (SP soil > WS soil > WG soil; Fig. S2A) whilst bacterial alpha diversity did not (Fig. S1A). Interestingly, despite being exposed to a less diverse fungal community in high-nutrient soils, plants recruited a similar number of fungal ASVs in all soil types (Fig S4A). However, for bacterial communities in the endosphere, alpha diversity decreased in plants grown in the high-nutrient SP soil (Fig. 5D), perhaps reflecting that bacterial belowground recruitment is more dependent on soil nutrition. Our results from these agricultural soils represent important confirmations of studies from controlled environments [57] and highlight the impact of modern agriculture practices on soil microbial community structure [58]. There are some recent studies that point to decreased ecosystem functioning in soils with reduced alpha diversity [59], although the long-term effects of soil fertilisation in plant production and resilience have yet to be fully examined. Our work suggests this intersection as one worthy of further study in seeking ways to develop more robust agriculture in the face of climate change.

Highly efficient symbiotic nodules were characterised by increased acquisition of iron and molybdenum, which are required as co-factors for the nitrogenase complex and for leghaemoglobin biosynthesis. The increase of expression of nicotianamine synthase, IMA peptides and MtMOT1.1 is linked to this (Fig. 6). Shoots of plants in symbiosis with high-efficiency WSM1022 rhizobia have higher levels of N, C, Mg, P, K, and Ca (Fig. 1C). We observed a significant reduction in total Mo accumulation in these shoots in comparison to mock-inoculated plants (Fig. 1C), despite the fact that WSM1022-inoculated plants are much larger (Fig. 2B–C). This suggests that when plants are engaged in highly efficient symbiosis, nodules are a stronger Mo sink than leaves, with the expression of Mo transport machinery in nodules regulated according to host-symbiont N fixation efficiency. Molybdenum and iron are necessary for the synthesis of the nitrogenase complex FeMo-cofactor that is necessary for symbiotic nitrogen fixation [60, 61]. Mo also acts as a co-factor for nitrate reductase [62]), thus Mo abundance in highly efficient nodules might reflect a switch from nitrate to ammonia metabolism in these organs. Moreover, iron is also required for leghaemoglobin biosynthesis, which is increased in higher efficiency nodules. We found three small secreted peptides (SSPs) belonging to the Iron-Man (IMA) family of proteins [37] which are responsible for the control of iron transport in plants and promote the uptake of iron into plant roots [63]. The induction of these genes is an indication of an increased nodule demand for these metal ions because of increased nitrogenase activity in efficient nodules. As well as increased SSP expression, highly efficient symbiotic nodules are also characterised by an increase of NIN- and SUNN- activated pathways as a reflection of higher levels of N being fixed and the subsequent activation of the AON response to inhibit further nodulation.

With higher expression in low-efficiency nodules, subcluster 2N included a number of NCR genes. NCRs are generally known to be involved in the nodulation process and bacteroid differentiation inside the symbiosome [64]; however, some are involved in the termination of the symbiotic partnership in non-efficient interactions [46] and some have been linked to the host allele-specific selection of rhizobial strains [65]. MtCLE35 (Medtr2g091125) and MtCLE34 (Medtr2g091120) are nodulation-suppressing genes induced by rhizobial infection and high nitrogen levels [66, 67]. The role of MtCLE34 in nodulation is poorly defined and in A17, it potentially contains a premature stop codon [67]; however, it has been little-studied. The NCR, CLE and SSP gene toolkits might act as part of the host machinery to fine-tune symbiosis, depending on if the rhizobial partner is high or low efficiency.

Symbiosis with the high-efficiency rhizobial strains WSM1022 and WSM419 brings the greatest benefit to plants in all soil types, but these benefits also vary depending on edaphic factors. The end benefits are also linked to the extent to which the endosphere microbial community impacts upon nodulation and the extent to which it is shaped alongside (and by) the active symbiosis in its midst. We found that the alpha diversity of the bacterial endosphere community decreased when plants were in symbiosis with high-efficiency nitrogen-fixing rhizobial strains (Fig. 5D). It was previously shown that the Shannon index of nodules is lower than roots, and we find enrichment in Actinobacterial ASVs classified as Pseudonocardiaceae, Mycobacteriaceae, Nocardia or Steptomycetaceae, in the endosphere of plants engaged in highly efficient N fixation. Actinobacteria have been linked to beneficial plant growth, possibly due to their antimicrobial action on other species [68] and they are known to be primary consumers of sugars, organic acids and amino acids in root exudates [69]. Therefore, it may be that the high-efficiency symbiotic state of plants alters plant fitness and root exudation, subsequently affecting bacterial endosphere colonisation (Fig. 7), which could lead to the community differences that we saw. This has been observed before in *Lotus japonicus* mutant plants that lost the ability to nodulate [18]. Root exudate composition is dependent on the N status and overall fitness of the plant [70–72], this in turn affects the composition of the rhizosphere and hence, the endosphere [73], leading to the types of shaping changes that we have found.

Symbiosis with the low-efficiency rhizobial partner Sm1021 led to an increase in dry weight, particularly on the WG and WS soils, but did not lead to an increase in macronutrient accumulation, instead trace minerals that are not linked to plant growth (Figs. 1C and 7). Moreover, nodulation with low-efficiency rhizobial strains triggered stress responses in the plant (including activation of nodule cell death responses) that are linked to nodule sanctioning and senescence [74] rather than leading to activation of nutrient uptake and effective usage. This response could underpin the alteration in the endosphere bacterial community observed between high- and low-efficiency strains. Increased abundance of Actinobacteria and Bacteroidetes (Fig. 5A) has been observed previously between domesticated and wild plant varieties, respectively [75]. The increased abundance of Actinobacteria could be a response to well-adapted, high-performing, domesticated varieties and high-efficiency rhizobial partnerships. The pathogen-like transcriptional response in low-efficiency symbiosis could suggest incapacity of low-efficiency rhizobial strains to “switch off” plant defence responses.

## Conclusions

By analysing not only plant and soil microbiome and nutrition but also plant growth and gene expression, we have characterised a mechanism for how soil impacts the outcome of symbiosis. We identified specific soil edaphic factors that correlate with bulk soil microbial community composition and suggest how those, plus rhizobial partner efficiency, shape the endosphere microbiome. Finally, we identified transcriptional changes that characterise high N fixation systems and that may account for the differential recruitment of endosphere microbial communities. These findings help to explain how highly efficient symbiosis may impact the soil and potentially soil health when legume crops are used in intercropping or field rotation. Overall, our findings highlight the importance of selecting the right inoculum for legume crops but one that is also right for the soil type and microbial community for optimal soil–plant–microbial interactions. Understanding this tri-partite association is thus crucial for crop improvement and for generating increased yield in a sustainable way. Analysis of the microbes identified from the endosphere in the high-efficiency symbiotic conditions will lead to a better understanding of the functional role of naturally co-occurring strains in soil. This could advance fine-tuning of microbial inoculums to significantly improve legume plant production.

## Methods

### Plant and soil materials

*Medicago truncatula* ecotype A17 seeds were used for all experiments. Wharf ground (WG) and Wick series (WS) soil types were taken from the University of Warwick Crop Centre site (Wellesbourne, Warwickshire, UK) and Spalding (SP) soil was taken from Jack Buck Farms (Spalding, Lincolnshire, UK); see Table S1 for soil characteristics including GPS locations of the collection sites.

### Plant growth in perlite pots

Seeds were extracted from the pods using a corrugated rubber mat and plasterer's hawk with handle, then scarified with concentrated sulphuric acid for 20–25 min. Seeds were then surface sterilised using a solution of 7% NaOCl for 5 min and washed 8 times with sterile water. Seeds were transferred to 1.5% water-agar plates and watered individually 3 times, leaving time between each application to allow the seeds absorb the water. Four layers of a plant growth pouch (CYG Growth Pouch, Mega International, USA) were used on the plate lid and watered above saturation since this increased the germination rate. Plates were sealed with micropore tape, covered with foil and stored upside down (agar up) at 4 °C for 5 days. Plates covered with foil were then transferred to a Sanyo 2279 growth cabinet for 2 days with 12/12 h light/dark, irradiance of 200 μmol m^−2^ s^−1^ and temperature of 24 °C (day) and 21 °C (night) (Fig. 1A). Successfully germinated seedlings (> 75%) were transferred to 11-cm pots (750 ml) with 85% autoclaved-sterile perlite at the bottom and 15% autoclaved sterile vermiculite at the top, watered with modified Broughton and Dilworth, 1970 nutrient solution prepared on reverse osmosis water without nitrogen (1 mM CaCl_2_·2H_2_O, 1 mM KH_2_PO_4_, 75 µM FeNaEDTA, 1 mM MgSO_4_·7H_2_O, 0.25 mM K_2_SO_4_, 6 µM MnSO4·H_2_O, 20 µM H_3_BO_3_, 1 µM ZnSO_4_·7H_2_O, 0.5 µM CuSO_4_·5H_2_O, 0.05 µM CoCl_3_, 0.1 µM Na_2_MoO_4_·2H_2_O, pH 6.2–6.4) and grown in a glasshouse compartment with a 16/8 h photoperiod (with artificial daylength extension) and temperature of 24 °C (day) and 21 °C (night). Pots and trays were sterilised with 75% ethanol; nutrient solution was applied on demand.

### Rhizobial culture preparation, plant inoculation and experimental design

Rhizobial strains *Sinorhizobium (ensifer) meliloti* 1021 (Sm1021), *Sinorhizobium (ensifer) meliloti* 1022 (WSM1022) and *Sinorhizobium (ensifer) medicae* 419 (WSM419) were kindly donated by Dr. Jason Terpolilli (Murdoch University, Australia). All strains were grown in pH-adjusted TY media (5 g/l tryptone, 3 g/l yeast extract, 6 mM CaCl_2_·2H_2_O, 4.766 g/l HEPES, pH 6.8, 12 g/l agar [76]) at 28 °C. Antibiotics were used for the appropriate growth of the rhizobial strains (streptomycin 100 µg/ml for Sm1021 and chloramphenicol at 20 µg/ml for WSM1022 and WSM419). Rhizobial strains were grown on plates for 2 days and a liquid culture was grown overnight. The day after transferring germinated seedlings to perlite pots, liquid cultures of rhizobia were spun at 2880 g for 7 min, washed with sterile water 3 times and resuspended in sterile water to an OD of 0.05; the same sterile water was used as mock (control) inoculation; 250 µl of the appropriate strain (mock, Sm1021, WSM419 or WSM1022) was applied to each plant by pipetting them around the root. Eleven days after inoculation (dai), perlite-grown plants were transferred to pre-watered soil pots. Plants in soil pots were watered with R0-deionised water from below (keeping the sterile matting wet) throughout the experiment. Plants were grown in a glasshouse compartment with a 16/8 h photoperiod (with artificial daylength extension) and temperature of 24 °C (day) and 21 °C (night). All material was sampled when plants were 66 days old (Fig. 1A). The whole experiment was carried out four times, running each biological repeat 1 week apart, with 5–8 plants per inoculation type.

### Sampling of plant material for RNAseq

Root samples of rhizobial-inoculated plants were removed from plants in perlite pots at 11 days after inoculation (dai) and snap-frozen in liquid N_2_ (Fig. 1A). Nodule samples were taken from plants in perlite pots at 21 dai, excised with a scalpel and snap-frozen in liquid N_2_ (Fig. 1A). The whole experiment was carried out three times, running each biological repeat 1 week apart; with 5–8 plants per inoculation type, these plants were pooled for one sequencing sample per experimental repeat. Samples were ground and RNA was extracted using the Monarch Total RNA Miniprep Kit (New England Biolabs). RNA sample quality was measured with a Bioanalyzer and quantity was calculated with Nanodrop and QuBit. Library preparation and RNAseq were performed by Novogene using Illumina HiSeq PE150.

### Sampling of plant material for phenotypic and mineral analysis

To evaluate the sampling point, two independent growth curves were run before the actual experiments; 49 dai was selected as the sampling time as plants had reached maximum growth but minimal flowering across all conditions was detected. Aboveground plant samples were taken from individual plants and weighed individually before being combined. For plant mineral analysis, all 8 biological repeats of a single experimental design were pooled together and homogenously ground using the Qiagen® Grinding Jar Set (Cat No./ID: 69,985) to a fine powder. The total content of C and N in plants was analysed by total combustion using a LECO Trumac® CN/N determinator; a ground 0.5-g sample (fine powder) was weighed into a ceramic boat and this was loaded into the instrument furnace (at 1350 °C).

### Sampling and analysis of soil

Input soil samples were taken at the time that each experimental repeat was set up (11 dai on the experimental design in Fig. 1A). The day before the sampling, photographs of the plants were taken for all biological repeats. On the day, aboveground plants were sampled into individual paper bags and oven-dried at 65 °C for 3 days before weighing.

For microbial community sampling, pots were turned upside down, soil in touch with the pot was removed, especially at the bottom of the pot. Bulk soil was sampled from a zone without roots; 2 pots were pooled in one lysing matrix E (MP Biomedicals) tube. Soil was removed until only the closest layer to the main core root remained, then two roots were pooled together into one tube. Samples were stored in a Falcon tube and transferred to a sterile hood where the tubes were filled with sterile water and vortexed so the rhizosphere soil would be washed off the roots. Roots were transferred to a fresh Falcon tube and rhizosphere soil solution was centrifuged at 4000 g for 15 min at RT. The supernatant was discarded and the soil pellet was sampled as rhizosphere-enriched soil (from now on, designed as ‘rhizosphere soil’). Roots were washed twice with 25 ml 0.1% Triton X-100 by vigorously shaking the tube then pouring it off. Roots were then washed once with sterile water, partially dried on sterile Whatman paper, cut into 1–3 mm pieces with sterile scissors and a representative sample was taken in a Lysing Matrix E (MP Biomedicals) tube. The endosphere sample was composed of roots and nodules.

For soil mineral analysis, equal samples of bulk soil from all pots of a single experimental repeat were pooled into one aluminium tray and oven-dried at 80 °C overnight. The complete soil sample was sieved through a 2-mm sieve (for pH and conductivity measurements and anion extraction) and half of it was ground with mortar and pestle to a fine powder (for ICP and total C and N analysis). pH and conductivity were measured from 2-mm soil samples incubated with distilled water (5 g in 12.5 ml) after shaking for 30 min at RT using a combined pH electrode. Soil texture and organic matter determination were carried out by NRM, UK. The total content of C and N in soil was analysed by total combustion using a LECO Trumac® CN/N determinator; a ground 0.5-g sample (fine powder) was weighed into a ceramic boat and this was loaded into the instrument furnace (at 1350˚C).

### Plant and soil sample preparation for ICP-MS

Soil samples for mineral analysis were taken by pooling bulk soil from all 8 pots from each experimental repeat; 0.2 g of plant material (fine powder) was weighed directly into digestion vessels. Six millilitres of concentrated HNO_3_ was added to each sample and microwave digestion was performed as below. Once digestion was complete, a solution was made to final volume of 20 ml with milliQ water; 0.2 g of dry soil (fine powder) was weighed directly into block digester tubes, 4 ml of HNO_3_ added, mixed and samples were run overnight (30 °C-30 min, 50 °C-1 h, 80 °C-14 h, 30 °C-∞]. The next day, 2 ml of HNO_3_ and 1 ml of HClO_4_ were added and tubes were run overnight on programme 2 (80 °C-8 h, 100 °C-2 h]. Then, 2.5 ml of HF was added, and samples were run on programme 3 (120 °C-1 h, 140 °C-3 h, 160 °C-4 h, 50 °C-∞). On the final day, the temperature was set to drop to 50 °C, then 2.5 ml of HNO_3_ and 2.5 ml of milliQ water were added and samples were left at 50 °C for 1 h. Cooled samples were adjusted to 50 ml with milliQ water in plastic volumetric flasks.

### Multi-elemental analysis by ICP-MS

Multi-element analysis of diluted solutions was carried out using ICP-MS (Thermo-Fisher Scientific iCAP-Q; Thermo Fisher Scientific, Bremen, Germany). The instrument was run employing three operational modes, including (i) a collision cell (Q cell) using He with kinetic energy discrimination (He-cell) to remove polyatomic interferences, (ii) standard mode (STD) in which the collision cell is evacuated and (iii) hydrogen mode (H_2_ cell) in which H_2_ gas is used as the cell gas. Samples were introduced from an autosampler (Cetac ASX-520) incorporating an ASXpress™ rapid uptake module through a PEEK nebuliser (Burgener Mira Mist). Internal standards were introduced to the sample stream on a separate line via the ASXpress unit and included Ge (10 µg/l), Rh (10 µg/l), and Ir (5 µg/l) in 2% trace analysis grade (Fisher Scientific, UK) HNO3. External multi-element calibration standards (Claritas-PPT grade CLMS-2 from SPEX Certiprep Inc., Metuchen, NJ, USA) included Ag, Al, As, Ba, Be, Cd, Ca, Co, Cr, Cs, Cu, Fe, K, Li, Mg, Mn, Mo, Na, Ni, P, Pb, Rb, S, Se, Sr, Tl, U, V and Zn, in the range 0–100 µg L-1 (0, 20, 40, 100 µg/l). A bespoke external multi-element calibration solution (PlasmaCAL, SCP Science, France) was used to create Ca, Mg, Na and K standards in the range 0–30 mg/l. P, B and S calibration utilised in-house (University of Nottingham) standard solutions (KH_2_PO_4_, K_2_SO_4_ and H_3_BO_3_). In-sample switching was used to measure B and P in STD mode, Se in H_2_ cell mode and all other elements in He cell mode. Sample processing was undertaken using Qtegra™ software (Thermo-Fisher Scientific) utilising external cross-calibration between pulse-counting and analogue detector modes when required. Tomato leaves (Standard Reference Material® SRM 1573a, National Institute of Standards & Technology (NIST), USA) and Montana II Soil (SRM 2711a, NIST-USA) were used as a reference for the plant and soil mineral analysis, respectively.

### Soil sample preparation and ion chromatography

Two grammes of soil as a fine powder was weighed in a Falcon tube and 20 ml of milliQ water added. Tubes were shaken overnight at RT. Samples were then centrifuged at 2000 rpm for 10 min. The supernatant was filtered through a 0.2-μm filter and stored at 4 °C until analysed. Fluoride, chloride, nitrate, phosphate and sulphate were analysed using a Thermo Scientific Dionex ICS-1100 ion chromatography system. Samples were analysed using a Na_2_CO_3_-NaHCO_3_ eluent at 1.4 ml/min, 28 mV (suppressor voltage) and a set temperature of 30 °C (column heater).

### Soil, rhizosphere and endosphere microbial DNA extraction, amplicon generation, library preparation and sequencing

DNA was extracted with MP Biomedicals FastDNA soil kit as per manufacturer’s instructions after grinding in Lysing matrix E (MP Biomedical) tube using a FastPrep (MP Biomedical) instrument. DNA was amplified with taxa specific primers for bacterial 16S (341f/785r (region V3-V4)) with 341f 5’–CCTACGGGNGGCWGCAG and 785r 5’–GACTACHVGGGTATCTAATCC [77] and for fungal ITS2, with ITS4 5’–GTGAATCATCGAATCTTTGAA and ITS86F 5’–TCCTCCGCTTATTGATATGC [78]. Amplification was performed using Q5® Hot Start High-Fidelity DNA Polymerase (NEB) with 53 °C annealing, 25 cycles and 55 °C annealing, 35 cycles for bacteria and fungi, respectively, with an extension time of 20 s for both. PCR products were cleaned using Agencourt AMPure XP magnetic beads (Beckman Coulter) and 80% EtOH wash. Sequencing indexes were added by PCR with Q5® Hot Start High-Fidelity DNA Polymerase (NEB); 55 °C, 15 s elongation and 8 cycles. Completed libraries were cleaned and normalised by SequalPrep normalisation plate (ThermoFisher). Libraries were pooled and sequenced by Illumina MiSeq paired-end 2 × 300 bp sequencing.

### Microbial sequence processing for bulk soil, rhizosphere and endosphere samples

DNA was extracted from all samples collected, i.e. 4 biological replicates (two pooled pots) for each sample. Prior to library preparation, biological replicates of DNA extracts from experimental repeats 1, 2 and 3 were combined in an equimolar manner for endosphere datasets from all three rhizobial strains. In experiments considering only WSM1022 vs. mock, biological replicates were computationally combined after rarefication (described below), yielding one sample per experimental repeat. The DADA2 pipeline [79] was used for initial quality trimming, error rate estimation, merging and community data matrix construction. For bacterial library filtering and trimming, a truncation quality score (TrunQ) of 10 was used, primer sequences from the start of reads removed and all reads truncated to a uniform length of 243 bp and 169 bp (forward and reverse, respectively). For fungal libraries, the same process was performed; TrunQ = 10 and lengths were 219 bp and 160 bp, respectively. Merged forward and reverse fungal sequences and bacterial forward reads only were used in further analysis of bulk soil and rhizosphere samples; reverse bacterial reads were of poor quality and were removed from our analysis. For endosphere samples of all three rhizobial strains, merged forward and reverse reads were taken forward.

Taxonomy assignment to the genus level was conducted using the UNITE ITS v8 UNITE Community (2019): UNITE general FASTA release for Fungi Version 18.11.2018 UNITE Community [80] and SILVA v132 formatted for DADA2; SILVA taxonomic training data formatted for DADA2 (Silva version 132); Zenodo [81]. The count of any reads found in mock sequencing samples, having been through the sequencing library construction process without DNA template input (de-ionised water) only), was considered contamination and subtracted from all other samples. ASVs designated as chloroplast or mitochondria were removed from all bacterial samples. Using a local BLAST search, all ASVs were checked for similarity to the plant host genome and removed if sequence similarity was greater than 90% over 90% of the query length. Sequences with very low abundance sequence starts (leading 4 bp) were also removed to improve analytical robustness (cumulatively < 1% of all reads). All libraries were rarefied to 4000 reads for experiments considering mock vs. WSM1022 only. For experiments considering endosphere samples for all strains, prior to rarefication, all sequences designated as genus Ensifer were catalogued and removed to avoid biasing analysis of differential ASVs because of the inoculum. Fungal reads were rarefied to 9116 and bacterial reads to 936. Some samples had extremely low reads after trimming and were removed before rarefication. On the basis of PCA (as described below), mock vs. WSM1022 samples from bulk soil and rhizosphere from experimental repeat 1, all clustered together and so were removed from further analysis. AVSs present at abundance above 0.1% across all soil samples were considered for analysis across soils and differential abundance analysis was carried out using DEseq2 [82] and the associated phyloseq extension ‘phyloseq_to_deseq2’ [83] as described in [84].

### Ecological indices and statistical analyses

Alpha diversity measures were calculated using the phyloseq package [83] and associated statistics were calculated using ANOVA. Normal distributions and equal variance assumptions were verified using Shapiro–Wilks and Bartlett tests, respectively. Beta diversity was calculated again using the phyloseq package with a Bray–Curtis distance metric and statistical calculations were made by PERMANOVA with 100 permutations using the vegan package [85]. Differential abundance was calculated using DEseq as described in [84]. Hierarchical clustering using the factoextra package in R [86] was used to delineate clusters of ASVs defining particular soil types. CCA analyses using the vegan package were performed with only soil nutrient data for bulk soil microbial communities, only plant aboveground nutrient data for endosphere microbial communities, and both aboveground plant and soil nutrient data for rhizosphere microbial communities. CCA coordinates and associated statistics were calculated (with 999 permutations for the latter) using the vegan package. Trimming of the reads was carried out to obtain the most comprehensive match between ASVs identified in different sequencing runs. The read start position between the two runs and bacterial ASVs from the first sequencing run had a uniform length of 243 bp. Bacterial ASVs from the second run were truncated to match this uniform length in order to obtain the best and least biased match between runs.

### Plant RNAseq data analysis

All samples collected for plant RNAseq analysis were submitted for library generation by Novogene for HiSeq Illumina PE150 sequencing. These reads were then trimmed using Trimmomatic software and quality checked. This generated an average of 24.77 M paired trimmed reads of 297 bp average length per sample. Splice aware mapping was carried out using STAR software to map reads onto the *Medicago truncatula* Mt4.0 reference genome and transcriptome to ensure accuracy of alignment and correct labelling of reads. This uniquely mapped 89.81% of reads. The resulting aligned sequences were outputted as SAM files and were converted into BAM files and sorted by index. Count files of the number of reads associated with genes were also produced. All three sequencing replicates and two replicates, respectively, were used for differential expression analysis of whole roots and nodules. On the basis of PCA clustering one of the nodule replicates was removed as an outlier. The R package DEseq2 was used to normalise and perform differential gene expression analysis (*P* < 0.05 after Benjamini–Hochberg correction) on the count files in order to produce a DESeqDataSet (dds) table which was then transformed with a regularized transformation (rlog) to normalise with respect to library size and account for small numbers of counts. PCA plots were generated using the R packages ggplot and ggplot2. Heatmaps displaying differentially expressed genes and their relative expression levels were generated using the R packages pheatmap, dplyr and ggplot. Cluster analysis was performed on the resulting heatmaps and optimal cluster number determined using the total within-cluster sum of square (elbow method). GO term, protein domain and pathway enrichment (*P* < 0.05 after Holm–Bonferroni correction) were carried out on clusters using the Phytomine tool on Phytozome.

### Soil and plant statistical analysis

Soil and plant mineral data was in transformed for normality (tested with a Shapiro–Wilk test) and statistically significant differences were detected with an ANOVA test followed by a Tukey post-hoc test. For mineral analyses, mean values were corrected using the mean dry weight of the plants per inoculation and soil type to calculate total element concentrations *in planta*. Plant dry weight data were tested for normality and variance; as the data was not normally distributed or had the same variances in all cases, a pairwise Wilcoxon test was performed to detect statistically significant differences. Values for all edaphic factors (Table S1) were linearly normalised across all samples by calculating z-scores and used for statistical analyses. Hierarchical clustering using the total within-cluster sum of square (elbow method) was used to group soil edaphic factors according to their occurrence.

## Supplementary Information

**Additional file 1: Figure S1.** Alpha and beta diversity and dominant bacterial taxa in Input and bulk soil samples (mock and WSM1022 inoculated). A. Alpha diversity. B. Beta diversity calculated using Bray-Curtis distance. C. Dominant bacterial taxa. For all, *n* = 3 pooled samples of 8 pots.

**Additional file 2: Figure S2.** Alpha and beta diversity and dominant fungal taxa in Input and bulk soil samples (mock and WSM1022 inoculated). A. Alpha diversity. B. Beta diversity calculated using Bray-Curtis distance. C. Dominant fungal taxa. For all, *n* = 3 pooled samples of 8 pots.

**Additional file 3: Figure S3.** Rhizosphere bacterial communities are shaped by rhizobial-inoculant, impacting plant nutrition. CCA of beta diversity of rhizosphere bacterial communities in rhizosphere soils from mock and WSM1022 inoculations, with the clusters of plant shoot nutrient factors from Fig. 1C (blue arrows); hash symbols denote significant correlation. CCA2 vs CCA3 of data in Fig. 4C. For all, *n* = 3 pooled samples of 8 pots.

**Additional file 4: Figure S4.** Diversity indices for fungal endosphere communities inoculated with Sm1021, WSM419, WSM1022 or mock inoculation. A. Alpha diversities represented by Shannon index. B. Beta diversity shown by Bray PCoA. Individual plot points indicate pooled samples of 8 biological replicates.

**Additional file 5: Figure S5.** RNAseq gene expression analysis on whole roots 11 days after inoculation. Heatmap of differentially expressed (*P* < 0.05) (DEseq2 R) transcripts between whole root samples. Analysis of GO term, protein domain and pathway enrichment is also shown here.

**Additional file 6: Table S1.** Plant aboveground dry weight values and statistical analysis.

**Additional file 7: Table S2.** Percentages of microbial diversity variation explained by soil type, soil nutrition and plant nutrition.

**Additional file 8: Table S3.** RNAseq values and analysis for nodule and root samples.

**Additional file 9: Supplementary Data S1.** Soil location and characteristics; soil mineral analysis for input and experimentally-derived samples and cluster information for soil edaphic factors.

**Additional file 10: Supplementary Data S2.** Plant mineral analysis.

**Additional file 11: Supplementary Data S3.** Microbiome community analysis.

## Data Availability

The RNAseq datasets supporting the conclusions of this article are available in the NCBI SRA database (Whole Root RNAseq SUB12091251, Nodule RNAseq SUB12002280) and the microbial sequencing datasets are available in the NCBI SRA database (Bulk soil and Rhizosphere Mock vs. 1022 microbiome dataset SUB12091286, Endosphere only microbiome all strains SUB12094414). The datasets supporting the conclusions of this article are included within the article and additional Supplementary Tables and Datasets.
