# Supplementary material for: Rhizobial nitrogen fixation efficiency shapes endosphere bacterial communities and Medicago truncatula host growth
Source: Microbiome. 2023 Jul 3;11:146. doi: 10.1186/s40168-023-01592-0 (PMC10316601; doi:10.1186/s40168-023-01592-0)
Supplement: Supplementary file 2 — Additional file 1: Figure S1. Alpha and beta diversity and dominant bacterial taxa in Input and bulk soil samples (mock and WSM1022 inoculated). A. Alpha diversity. B. Beta diversity calculated using Bray-Curtis distance. C. Dominant bacterial taxa. For all, n = 3 pooled samples of 8 pots. [file 40168_2023_1592_MOESM1_ESM.pdf]

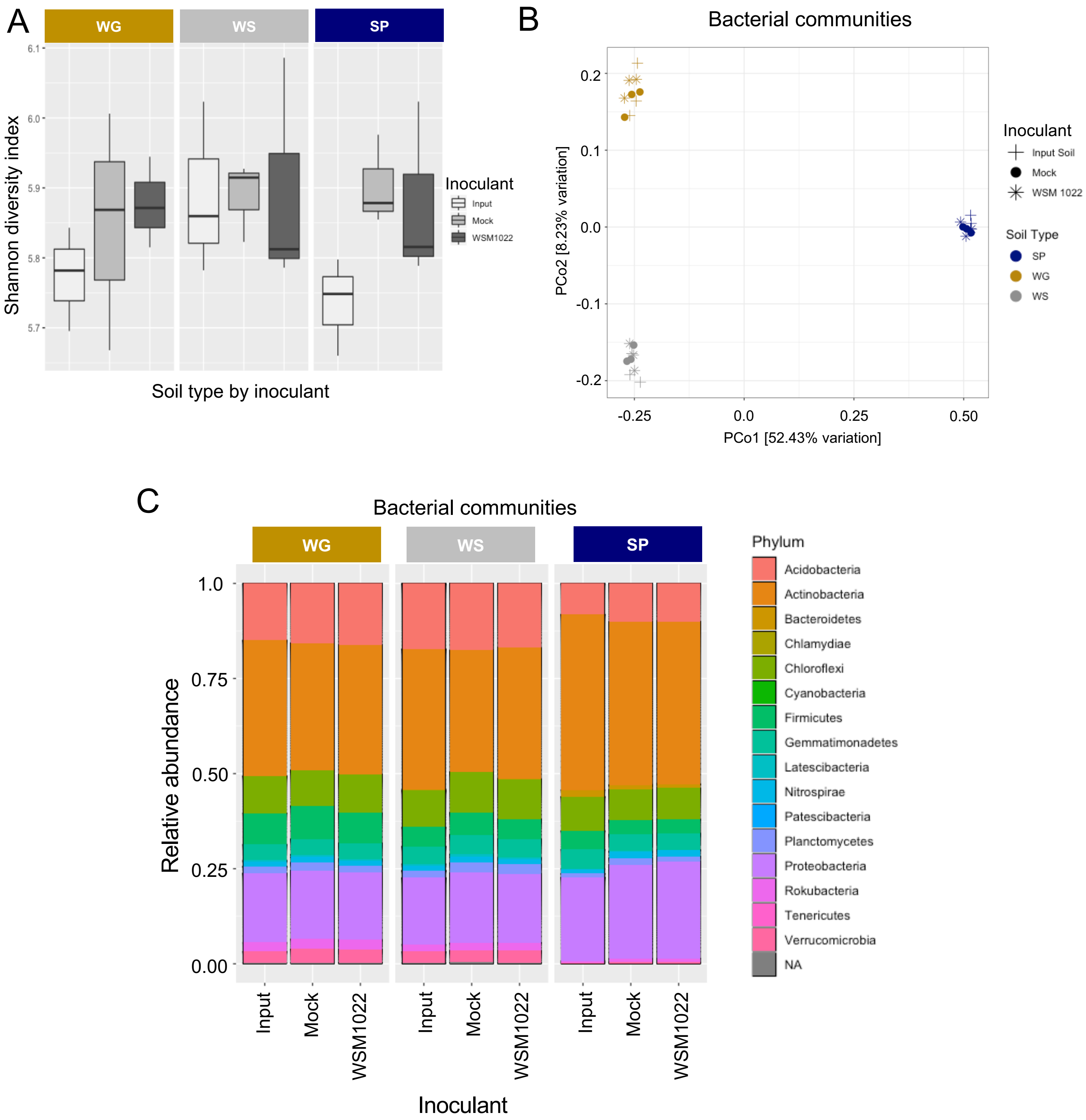

Figure S1. Alpha and beta diversity and dominant bacterial taxa in Input and bulk soil samples (mock and WSM1022 inoculated). A. Alpha diversity. **B**. Beta diversity calculated using Bray-Curtis distance. **C**. Dominant bacterial taxa. For all, n=3 pooled samples of 8 pots.
