# Supplementary material for: Rhizobial nitrogen fixation efficiency shapes endosphere bacterial communities and Medicago truncatula host growth
Source: Microbiome. 2023 Jul 3;11:146. doi: 10.1186/s40168-023-01592-0 (PMC10316601; doi:10.1186/s40168-023-01592-0)
Supplement: Supplementary file 4 — Additional file 3: Figure S3. Rhizosphere bacterial communities are shaped by rhizobial-inoculant, impacting plant nutrition. CCA of beta diversity of rhizosphere bacterial communities in rhizosphere soils from mock and WSM1022 inoculations, with the clusters of plant shoot nutrient factors from Fig. 1C (blue arrows); hash symbols denote significant correlation. CCA2 vs CCA3 of data in Fig. 4C. For all, n = 3 pooled samples of 8 pots. [file 40168_2023_1592_MOESM3_ESM.pdf]

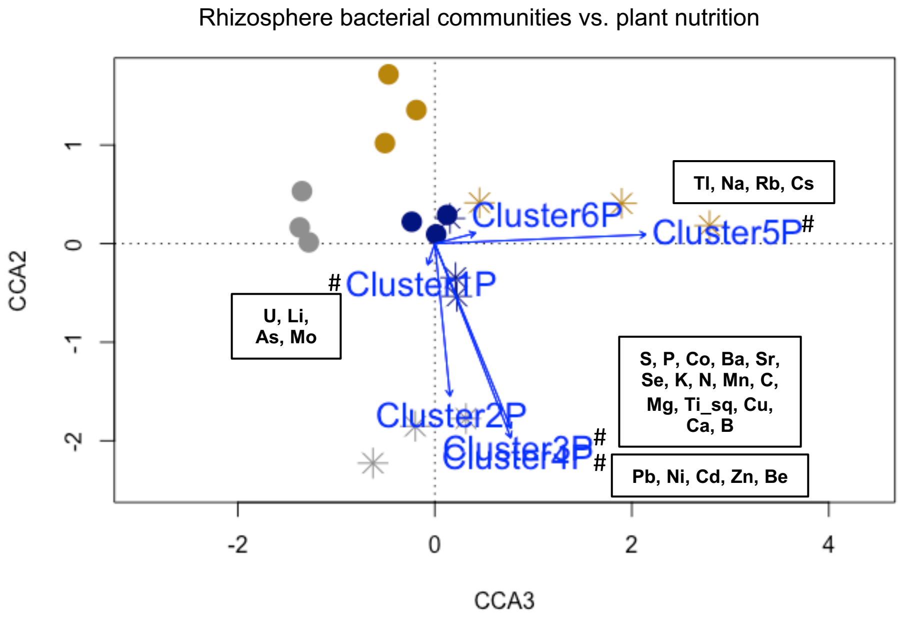

Figure S3. Rhizosphere bacterial communities are shaped by rhizobial-inoculant, impacting plant nutrition. CCA of beta diversity of rhizosphere bacterial communities in rhizosphere soils from mock and WSM1022 inoculations calculated using Bray-Curtis distance, with the clusters of plant shoot nutrient factors from Fig. 1C (blue arrows); hash symbols denote significant correlation. CCA2 vs CCA3 of data in Fig. 4C. For all, n=3 pooled samples of 8 pots.
