# Supplementary material for: Rhizobial nitrogen fixation efficiency shapes endosphere bacterial communities and Medicago truncatula host growth
Source: Microbiome. 2023 Jul 3;11:146. doi: 10.1186/s40168-023-01592-0 (PMC10316601; doi:10.1186/s40168-023-01592-0)
Supplement: Supplementary file 5 — Additional file 4: Figure S4. Diversity indices for fungal endosphere communities inoculated with Sm1021, WSM419, WSM1022 or mock inoculation. A. Alpha diversities represented by Shannon index. B. Beta diversity shown by Bray PCoA. Individual plot points indicate pooled samples of 8 biological replicates. [file 40168_2023_1592_MOESM4_ESM.pdf]

A

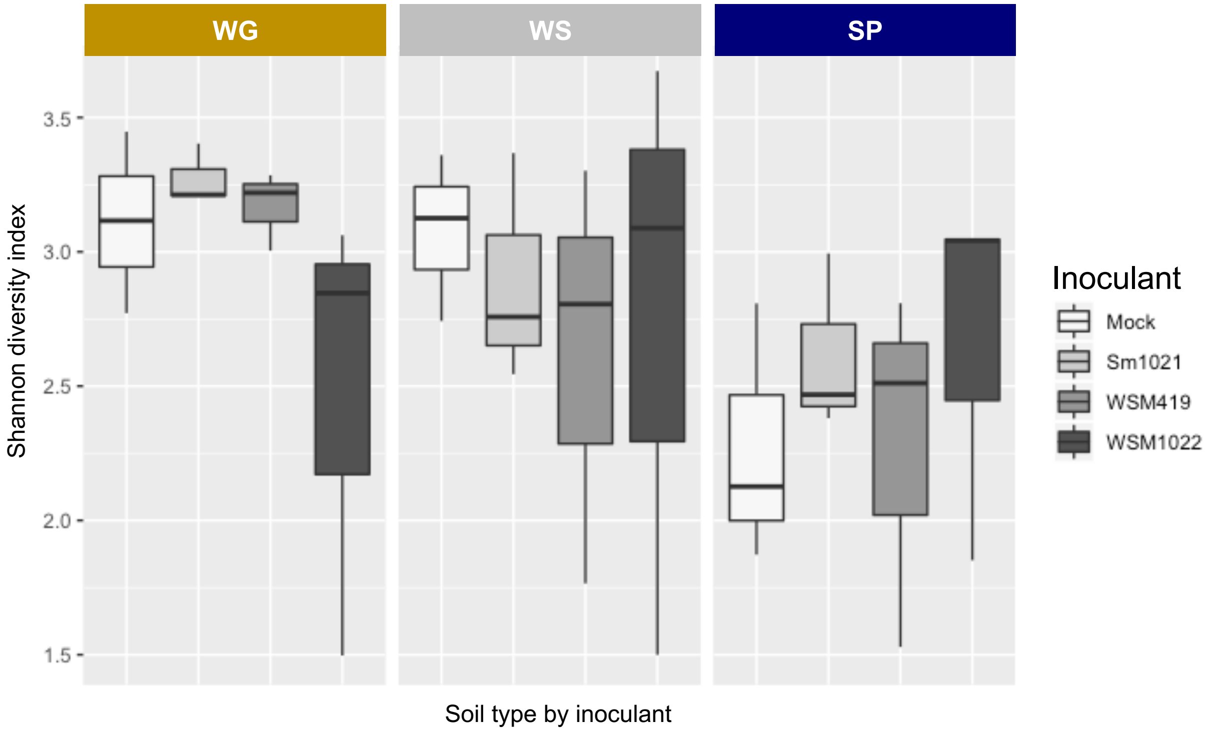

B

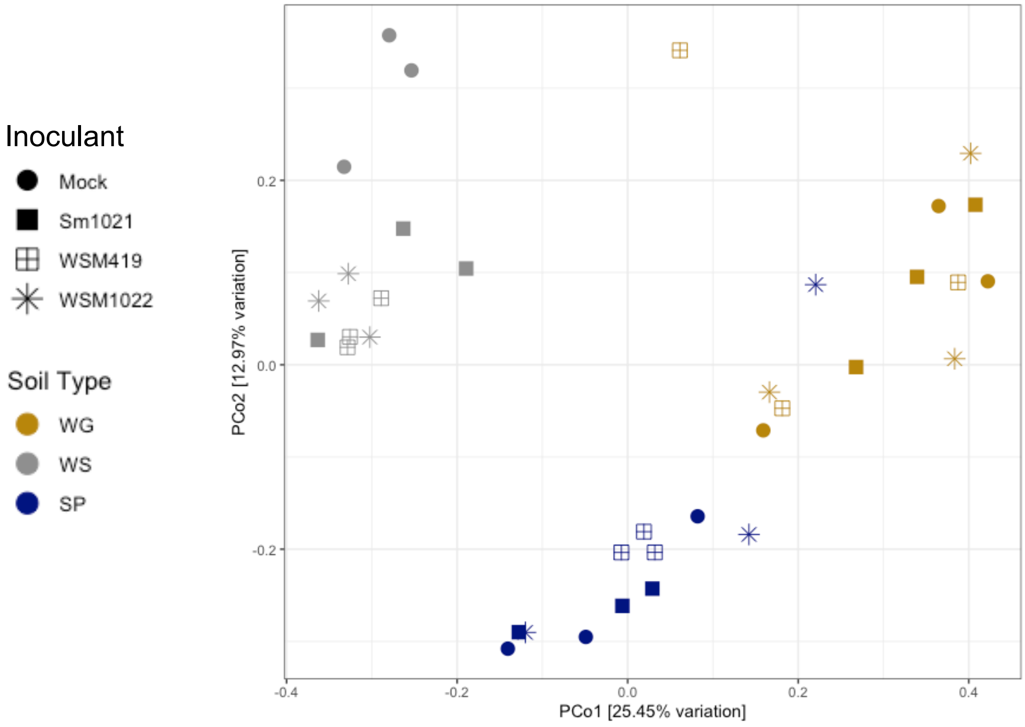

Figure S4. Diversity indices for fungal endosphere communities inoculated with Sm1021, WSM419, WSM1022 or mock inoculation. A. Alpha diversities represented by Shannon index. **B.** Beta diversity shown by Bray PCoA. Individual plot points indicate pooled samples of 8 biological replicates.
