# Supplementary material for: Rhizobial nitrogen fixation efficiency shapes endosphere bacterial communities and Medicago truncatula host growth
Source: Microbiome. 2023 Jul 3;11:146. doi: 10.1186/s40168-023-01592-0 (PMC10316601; doi:10.1186/s40168-023-01592-0)
Supplement: Supplementary file 6 — Additional file 5: Figure S5. RNAseq gene expression analysis on whole roots 11 days after inoculation. Heatmap of differentially expressed (P < 0.05) (DEseq2 R) transcripts between whole root samples. Analysis of GO term, protein domain and pathway enrichment is also shown here. [file 40168_2023_1592_MOESM5_ESM.pdf]

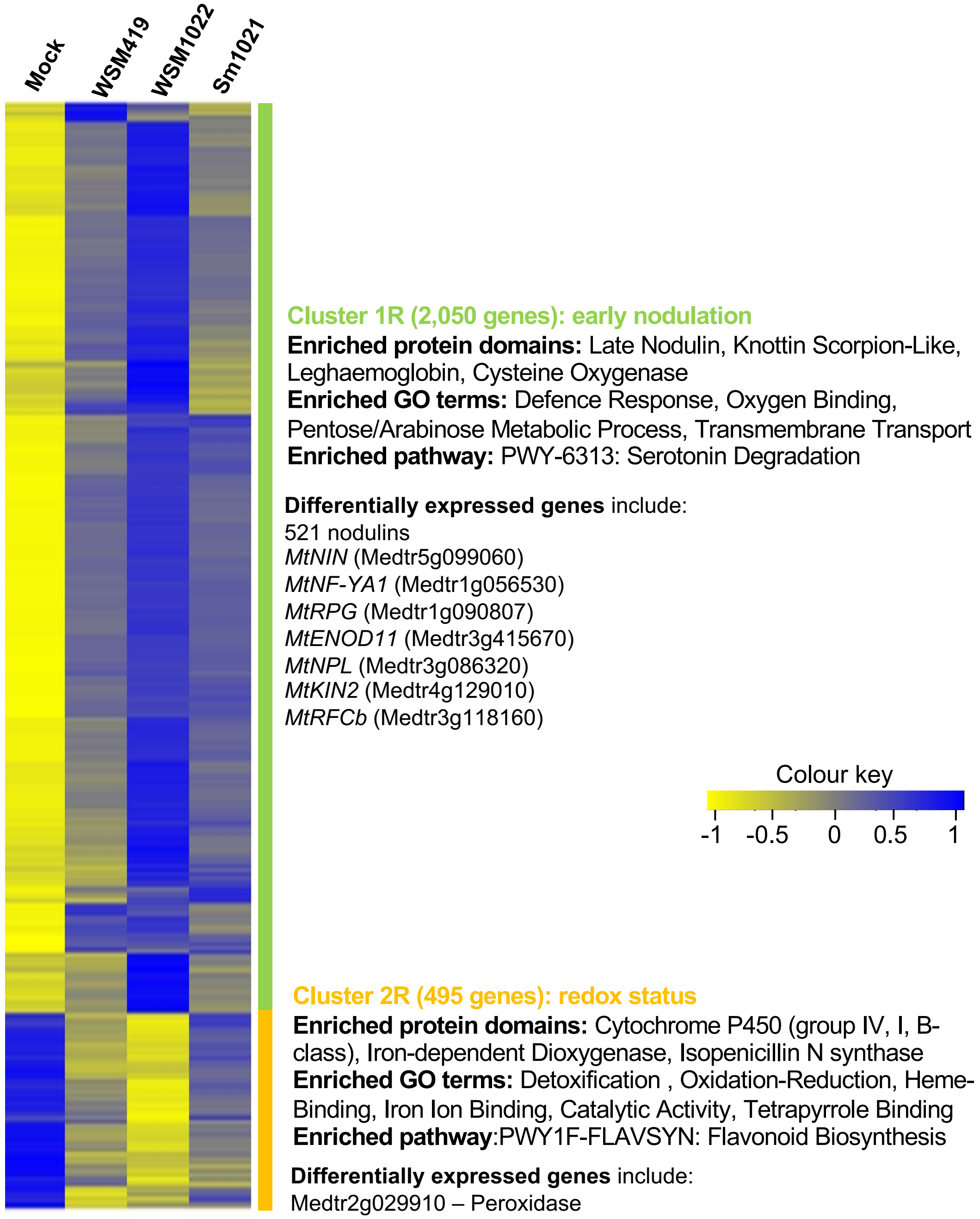

Figure S5. RNAseq gene expression analysis on whole roots 11 days after inoculation. A. Principal Component Analysis (PCA) plot of Medicago inoculated with one of the three rhizobial inoculants (Sm1021, WSM419, WSM1022) or mock-inoculated. **B.** Heatmap of differentially expressed (P<0.05) (DEseq2 R) transcripts between whole root samples. Analysis of GO term, protein domain and pathway enrichment is also shown here.
